# Discriminating between Medium-Sized Tridactyl Trackmakers: Tracking Ornithopod Tracks in the Base of the Cretaceous (Berriasian, Spain)

**DOI:** 10.1371/journal.pone.0081830

**Published:** 2013-11-26

**Authors:** Diego Castanera, Carlos Pascual, Novella L. Razzolini, Bernat Vila, José L. Barco, José I. Canudo

**Affiliations:** 1 Grupo Aragosaurus-IUCA, Paleontología, Facultad de Ciencias, Universidad de Zaragoza, Zaragoza, Spain; 2 Soria, Spain; 3 Institut Català de Paleontologia Miquel Crusafont, Sabadell (Barcelona), Spain; 4 Paleoymás. Polígono INBISA - Empresarium, Zaragoza, Spain; University of Pennsylvania, United States of America

## Abstract

**Background:**

Recent work on the Jurassic-Cretaceous transition of the Iberian Range (Spain) has opened a new window onto the interpretation of the trackmakers of some medium-sized tridactyl tracks. The ichnotaxon *Therangospodus oncalensis* has been described in the Huérteles Formation (Berriasian) and is one of the classical tracks from the area assigned to medium-sized theropods.

**Methodology/Principal Findings:**

A review of the type locality of *Therangospodus oncalensis* (Fuentesalvo tracksite) and other tracksites from the Huérteles Formation (Berriasian) has yielded new information on the morphology, gait and trackmaker identity of the aforementioned ichnospecies. The new data suggest that the trackmaker is an ornithopod rather than a theropod on the basis of the length/width ratio, the anterior triangle length-width ratio, the short steps, the round to quadrangular heel pad impression and the probable manus impressions.

**Conclusions/Significance:**

*T. oncalensis* shows similarities with various tracks from the Berriasian of Europe assigned to *Iguanodontipus*. The ichnotaxonomical status of this ichnospecies is here considered as *Iguanodontipus? oncalensis* due to the current state of knowledge of the ichnotaxonomy of medium-sized ornithopod tracks. This reassessment of *I? oncalensis* also has two significant implications for the palaeoecology of the faunas during the deposition of the Huérteles Formation: 1- the high number and percentage of theropod tracks would be lower than previous papers have suggested. 2- the gregarious behaviour described in the type locality (Fuentesalvo) would be among ornithopods instead of theropods.

## Introduction

When it comes to assigning a tridactyl track to a particular producer (theropod vs. ornithopod), controversy is common in the vertebrate ichnology of the Mesozoic (see 1-5). In the Huérteles Formation (Berriasian) there seems to be a notable disproportion between the number of theropod and ornithopod tracks [6,7]. This anomaly in the relative abundance of theropod/ornithopod tracks has been explained as a palaeoecological consequence of the greater activity of the theropods “typical of such predatory groups” [7]. Recent work on the Jurassic-Cretaceous interval has shown the difficulties in identifying the producer of small to medium-sized tridactyl tracks [8-13], so this disproportion could also be a matter of the misidentification of at least some of the ornithopod tracks. The ichnogenus *Therangospodus* was first used in the Huérteles Formation by Moratalla [14] in his doctoral thesis. Nonetheless, it was not until 1998 that it was formally erected by Lockley et al. [15]. In this latter paper, the authors included two ichnospecies within the ichnogenus: *Therangospodus pandemicus* from the Late Jurassic of North America and Asia, and *Therangospodus oncalensis* from the Early Cretaceous of Europe (though in the original paper they considered its age to be ?Late Jurassic-Early Cretaceous). Since then *Therangospodus* has been considered an ichnogenus of medium-sized theropods (15-30 cm in track length), and has been described in tracksites from Europe [16-18], North America [19,20] and Asia [21,22]. Other tracks that have been associated with *Therangospodus* on account of their resemblance have been described in the Late Jurassic of Italy [23] and Switzerland [24]; tracks attributed to *cf. Therangospodus* have been described in Argentina as well [25]. Furthermore, Barco et al. [16] report new tracks and trackways of *T. oncalensis* from the type tracksite that suggest gregarious behaviour. In all of these cases the tracks have been considered to be produced by theropods, but in the case of the Swiss tracks Marty [24] points out that their morphotype II is “similar to the ichnogenus *Therangospodus*, but on the other hand also shares many characteristic features of ichnogenera attributed to ornithopod dinosaurs”. Furthermore, this author suggests that according to the illustrations of Barco et al. [16] *Therangospodus oncalensis* also “shows a striking similarity with ornithopod tracks”.

Recent work in the Iberian Range of Spain [8–10,13] on the Las Cerradicas tracksite, which is roughly similar in age, has shown that at least some ornithopods could have produced theropod-like pes tracks. Even though these tracks have not been assigned to any particular ichnogenus, their resemblance to *Therangospodus* has been noted [13,26]. The Las Cerradicas tracksite has opened a new window onto the interpretation of tridactyl tracks during the Jurassic-Cretaceous interval, especially in Europe where similarly problematic tracks have been described in other tracksites [11,12]. One of the criteria for distinguishing between a theropod and an ornithopod trackmaker has been the presence of manus prints, which suggests a quadrupedal trackmaker. Nonetheless, there might be a manus preservation bias depending on the depth where the tracks are recorded (undertracks), such that the manus prints might seem to be absent [13]. Other typical characters that suggest an ornithopod origin are the length/width ratio, the anterior triangle length-width ratio, short steps and the inward rotation of the pes prints [8,10,27].

In the light of these problematic questions regarding the identity of the trackmakers of *Therangospodus oncalensis*, a review of the type tracksite of the ichnospecies (Fuentesalvo) and other tracksites from the same formation (Huérteles) has been carried out. The aim of this paper is thus to review the ichnotaxonomical affinities of *Therangospodus oncalensis*, describe new trackways here assigned to the same ichnospecies, and discuss some aspects of the trackways in terms of the gait and behaviour of the trackmakers.

### Geographical and Geological Setting

**Figure 1:**
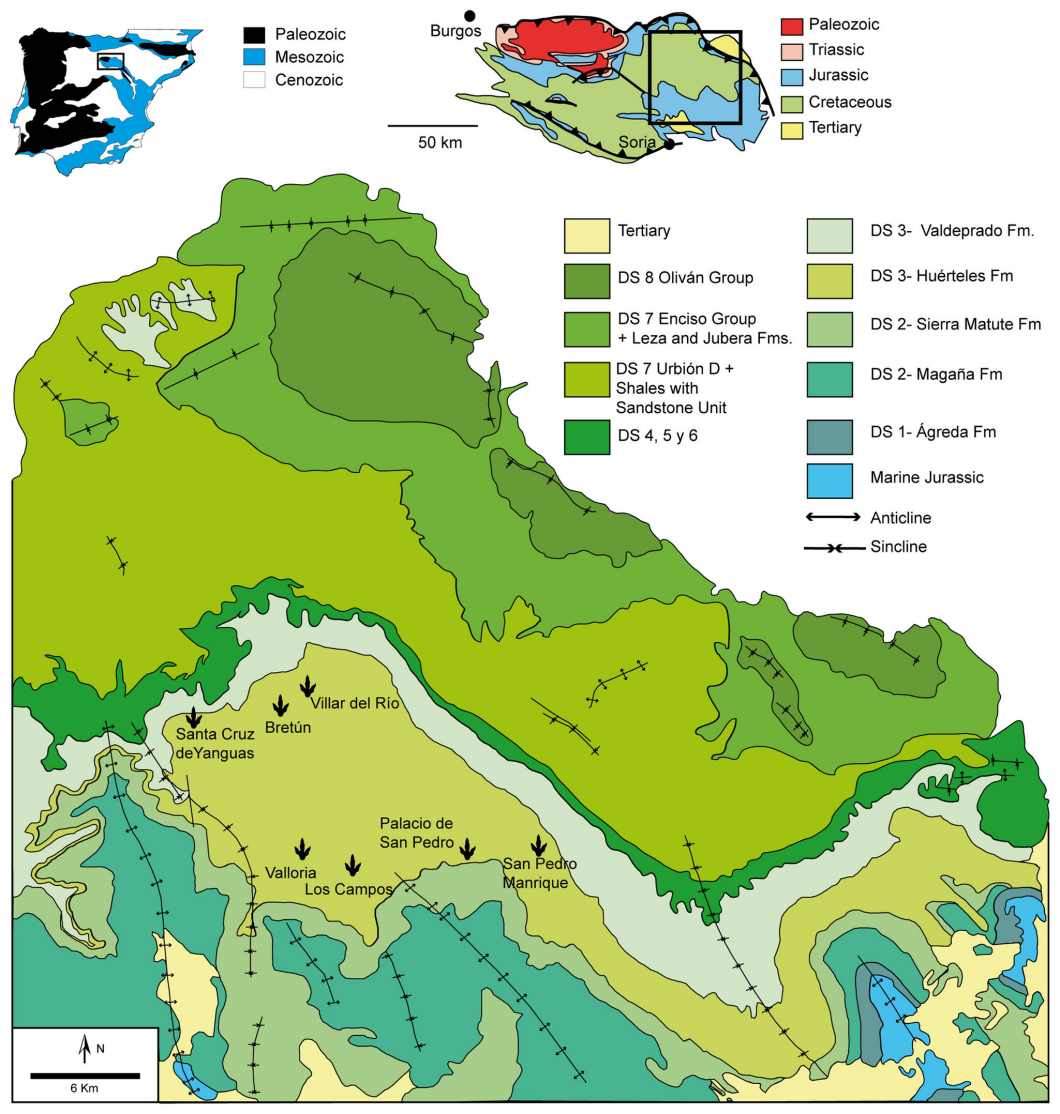
Geographical and geological setting. The tracks show the localities where the tracksites with *I?oncalensis* are located (modified from [7]).

The studied tracks come from the region of Tierras Altas (Figure 1) in the north of Soria province, north-central Spain. The studied tracksites are Fuentesalvo in the locality of Villar del Río, La Peña and Fuente Lacorte in the locality of Bretún, Los Tormos in the locality of Santa Cruz de Yanguas, Valdelavilla in the locality of San Pedro Manrique, Salgar de Sillas in the locality of Los Campos, Valloria IV in the locality of Valloria and La Losa II in the locality of Palacios de San Pedro. Geologically, all of them are located in the Huérteles Formation, which belongs to depositional sequence 3 of the infill of the Cameros Basin [28,29]. This was thought to have been deposited in alluvial plain systems distally connected with playa-lake systems ([7,30] and references therein), though recent works [31,32] are changing this palaeoenvironmental interpretation, the latter arguing that the Huérteles Formation was deposited in a tide-influenced fluvial-deltaic setting. The dinosaur tracksites are located in the proximal environments (sandy-muddy flats and mud flats according to Gómez-Fernandez and Meléndez [7,30]. The estimated age of the Huérteles Formation is Berriasian. This dating has been proposed on the basis of ostracods, charophytes and stratigraphic correlations [29,33,34].

## Materials and Methods

All necessary permits were obtained for the described study, which complied with all relevant regulations. The regional government (Junta de Castilla y León) provided the permits to document the ichnological tracksites. We reviewed the tracksites where *T. oncalensis* has been described or cited by previous authors [6,14,16,35,36] as well as others that remain undescribed. The acronyms used for each tracksite follow the aforementioned works and are: Fuentesalvo (FTS), La Peña (LP), Salgar de Sillas (SS), Valloria IV (VAIV), Los Tormos (LTR), Valdelavilla (VDV), Fuente Lacorte (FC), Valdecantos (VDC), La Losa II (LLII). The most interesting tracksites are Fuentesalvo and La Peña. In the former, 15 trackways (FTS1-FTS15) have been described [14–16] in the main surface of the tracksite, and another two (FTS16-FTS17) in the upper level. Furthermore, isolated tracks have been described as well (FTS0.1-FTS0.7). The erosion produced by the ravine has uncovered new, undescribed tracks that probably represent at least three trackways (FTS18-FTS20), though their interpretation is difficult. Aguirrezabala and Viera [35] and Moratalla [14] described one trackway (LP1) at La Peña. Two additional trackways (LP2 and LP3) have been discovered during the cleaning and preparation of the tracksite for tourist visits, and these are described below. In Los Tormos (level 2) Moratalla [14] cited the presence of *Therangospodus* but without describing any trackway in detail. Aguirrezabala and Viera [36] had described some of these tracks. During the topographical survey of the Tierras Altas region in 2004, the Paleoymás team differentiated at least four trackways (LTR5, LTR6, LT7, and LTR8) and other isolated tracks (LTR0.24, LTR0.25, LTR30 and LTR32) that are similar in general morphology. Likewise, in the Fuente Lacorte tracksite (level 10) at least five trackways (FC4, FC5, FC8, FC9, FC11) have been differentiated. One isolated trackway in Valdelavilla level VIII (VDV-VIII-1) has been described by Pascual and Sanz [37] and cited as *Therangospodus* by Hernández et al. [6]. At La Losa II one isolated trackway has been cited by Fuentes Vidarte et al. [38,39]. At Salgar de Sillas (SS26) and Valloria IV (VAIV1) there is one isolated trackway in each tracksite, as well as some isolated tracks in the former (SS27). Furthermore, specimen MNS-2002-96-2 stored at the Museo Numantino de Soria and figured by Fuentes Vidarte et al. [39] is also included in our analysis.

We took photographs of every isolated track to analyse the track morphology independently. In some of the tracks (FTS19, LP1.3, LP1.5, LP2.3, LP2.12, LP3.8, VAIV1.1, VAIV1.2, VAIV1.3, LTR0.32 and LII1.4) we constructed photogrammetric models using the software Visual SFM, Paraview and Meshlab (see 40). In the case of the La Peña tracksite, a map was also made, reticulating the tracksite in squares of 50 cm, taking perpendicular photographs of each square and then combining them using Adobe Illustratror software. The trackways from Fuentesalvo, Salgar de Sillas, Los Tormos and Fuente Lacorte were taken from the maps of the tracksites made during the topographical survey of Tierras Altas. The trackway from Valdelavilla level VIII was redrawn in accordance with Pascual and Sanz [37].

**Figure 2:**
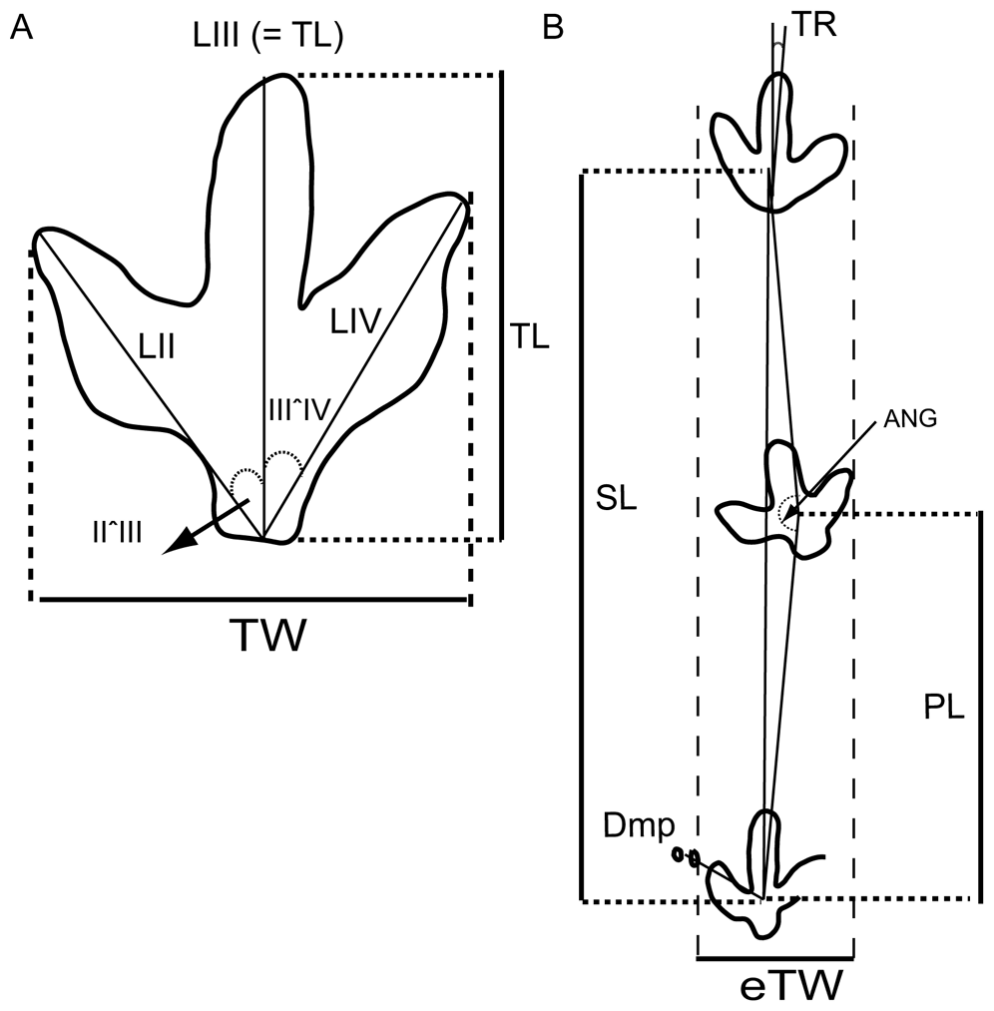
Measurements taken from the tracks. A) Measurements taken for the whole trackway. B) Measurements taken for the individual tracks. Abbreviations: see text in Materials and Methods.

The terminology used in this paper mainly follows the works of Thulborn [2] and Marty [24]. Measurements were taken (Figure 2, Appendix S1) for the footprint length (FL), footprint width (FW), manus–pes distance (Dm–p), length of the digits (LII, LIII, LIV), interdigital angles (II–III, III–IV), pace length (PL), stride length (SL), pace angulation (ANG), footprint rotation (FR), and external trackway width (eTW). The anterior triangle length-width ratio (AT) was calculated according to Lockley [8]. The data were collected from the field and from the original papers. The statistical analysis of the data was performed using the software PAST [41]. We mainly analysed the FL/FW ratio and the PL/FL ratio. We selected these parameters because these best represent the dimensions and the length of the steps in the tracks, and are two significant parameters in distinguishing theropod from ornithopod tracks [8,27]. We carried out a bivariate analysis of the FL/FW ratio and the PL/FL ratio using the average values for each trackway (Appendix S2). The data where the accuracy is uncertain (represented by ? in Appendix S1) have not been included in the average values. The data for the ichnotaxa plotted in these diagrams were taken from the literature and are shown in Appendix S3. Moreover, we have included the AT values in relation to the FL/FW ratio, as in the diagrams published by Lockley [8]. The AT (Appendix S2) for the described tracks at Fuentesalvo [16] has been calculated for one track (the best preserved) from each trackway. 

### Systematic ichnology

*Iguanodontipus? oncalensis*

Figures 3-11

1980 *Iguanodon* footprint [35], pictures 8, 9.

1983 *Iguanodon* footprint [36], picture 5.

1983 *Megalosaurus* footprint [36], picture 7.

1993 *Therangospodus oncalensis* nov. ichnosp. [14], p. 106, 189-202, figs. 8.5.1, 8.5.2, 8.6.1 (B), 8.6.2 (A). 

1998 *Therangospodus oncalensis* (Moratalla, 1993) Amended [15], p. 347-348, figs. 1B-C, 7.

2000 Theropod footprint [37], figs. 15, 20.

2002 Iguanodontid trackway [38], fig. 8.

2005 *Therangospodus oncalensis* [39], fig.9.

2005 Ornithopod footprint [39], fig. 15.

2005 Ornithopod trackway [39], fig. 17.

**Figure 3:**
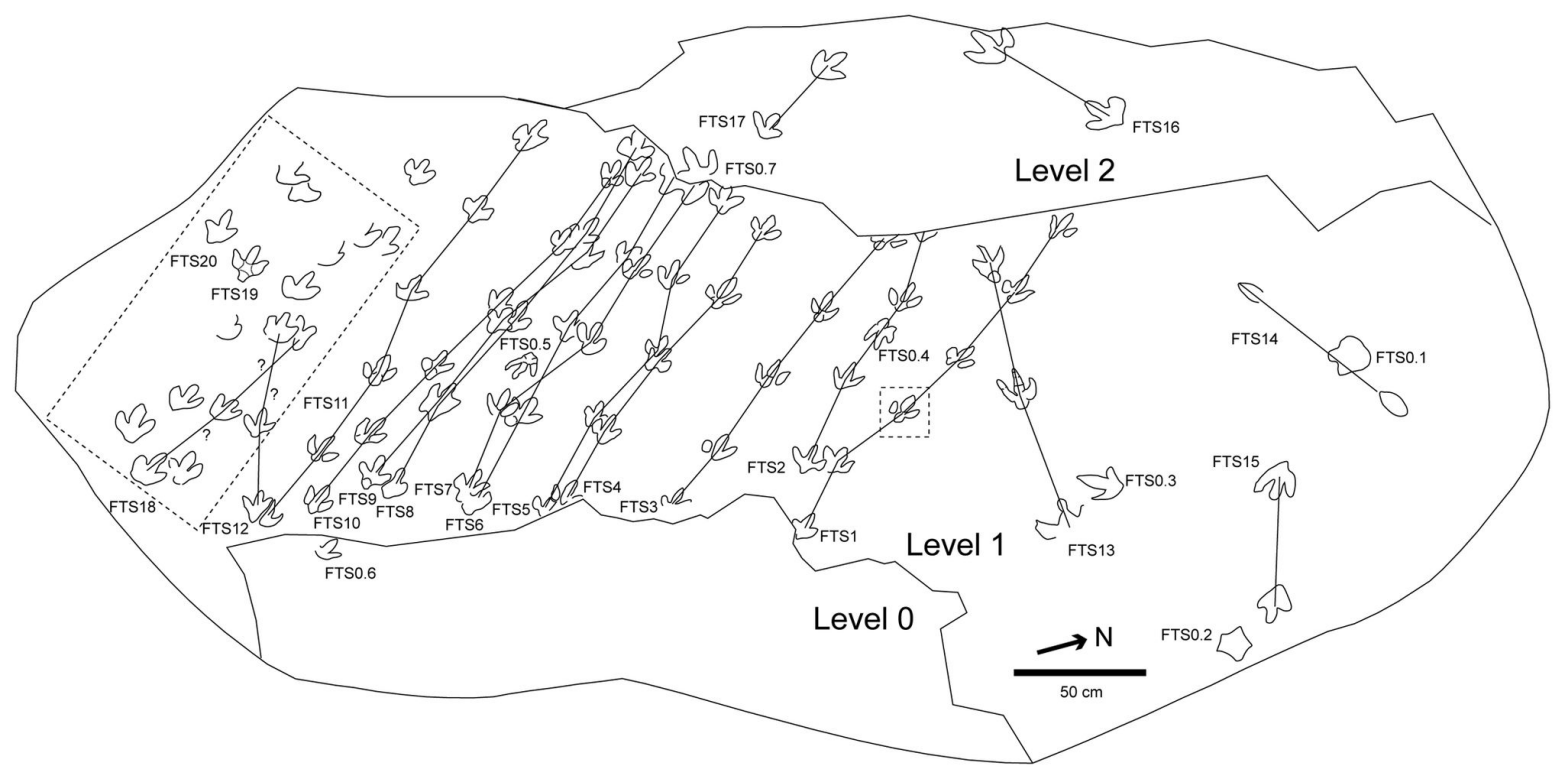
Sketch map of the Fuentesalvo tracksite (modified from [16]). The dotted lines indicate the undescribed part of the tracksite and track 1.3 from the holotype trackway (see figure 4).

**Figure 4:**
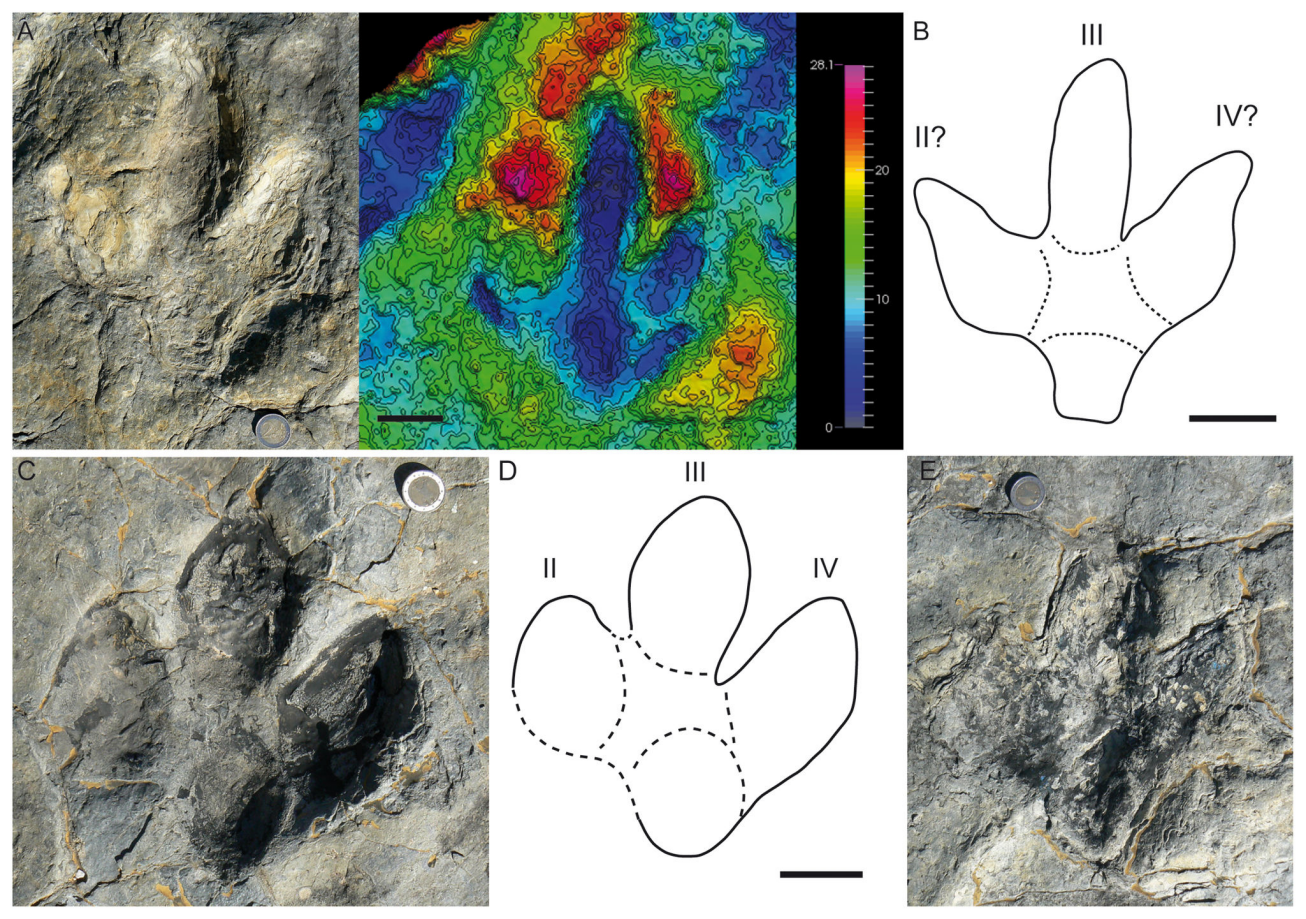
*I?oncalensis* tracks from the Fuentesalvo tracksite. A) Picture and photogrammetric 3D depth analysis model of the new track (FTS19). Note the three microlayers inside the track. B) Outline drawing of track FTS19. C) Pictures of track FTS1.3 from the holotype trackway without any evidence of the aforementioned microlayers. D) Outline drawing of the FTS1.3 from the holotype trackway. E) Picture of track FTS10.3 showing the microlayers. These tracks have been coloured in black with earthy tones. Note also the differences in the morphology of the heel pad impression (round to quadrangular) between the tracks. Scale depth in the models in mm. Scales: coin = 2.5 cm diameter; scale bar = 5 cm.

**Figure 5:**
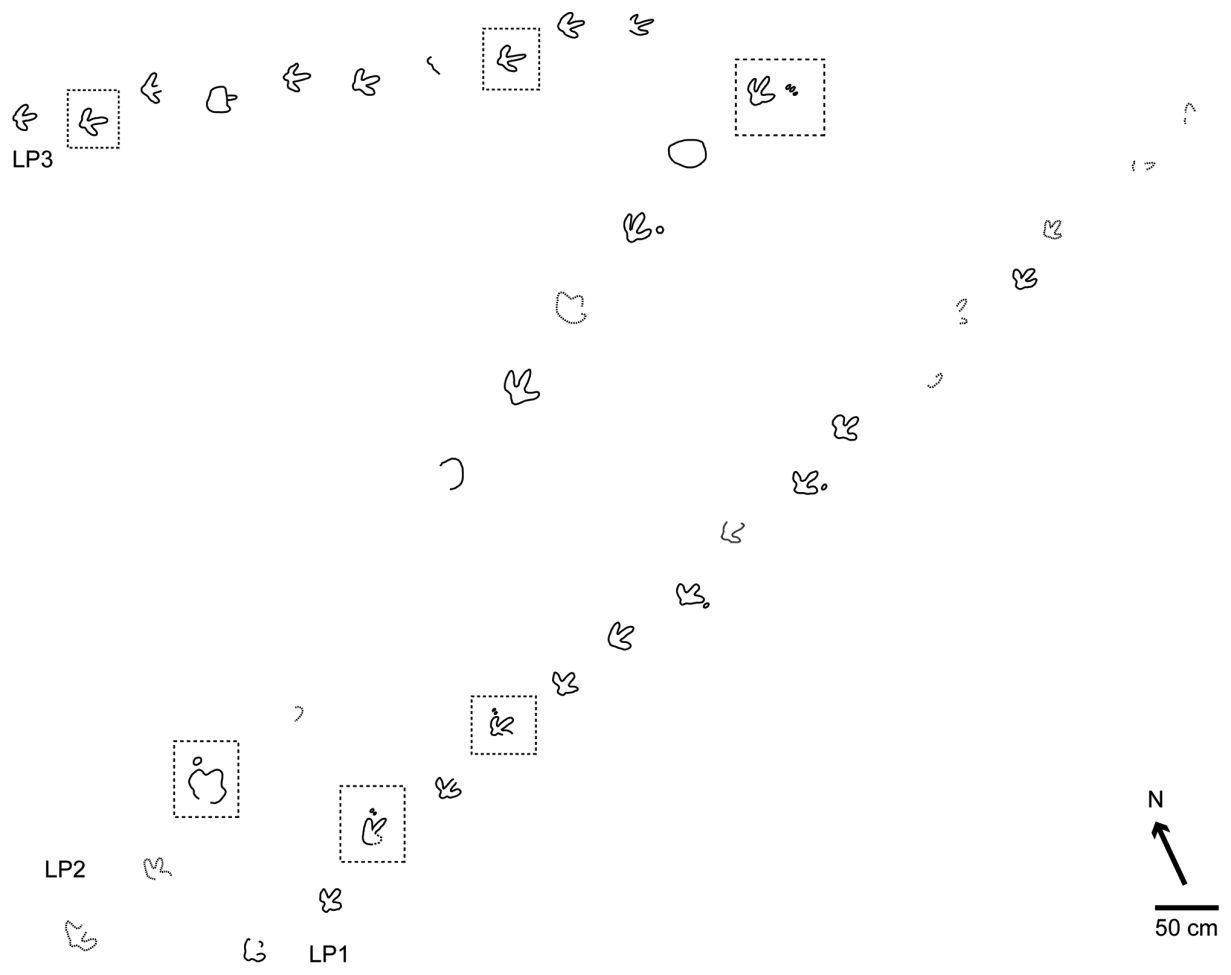
Sketch map of the La Peña tracksite (Bretún locality).

**Figure 6:**
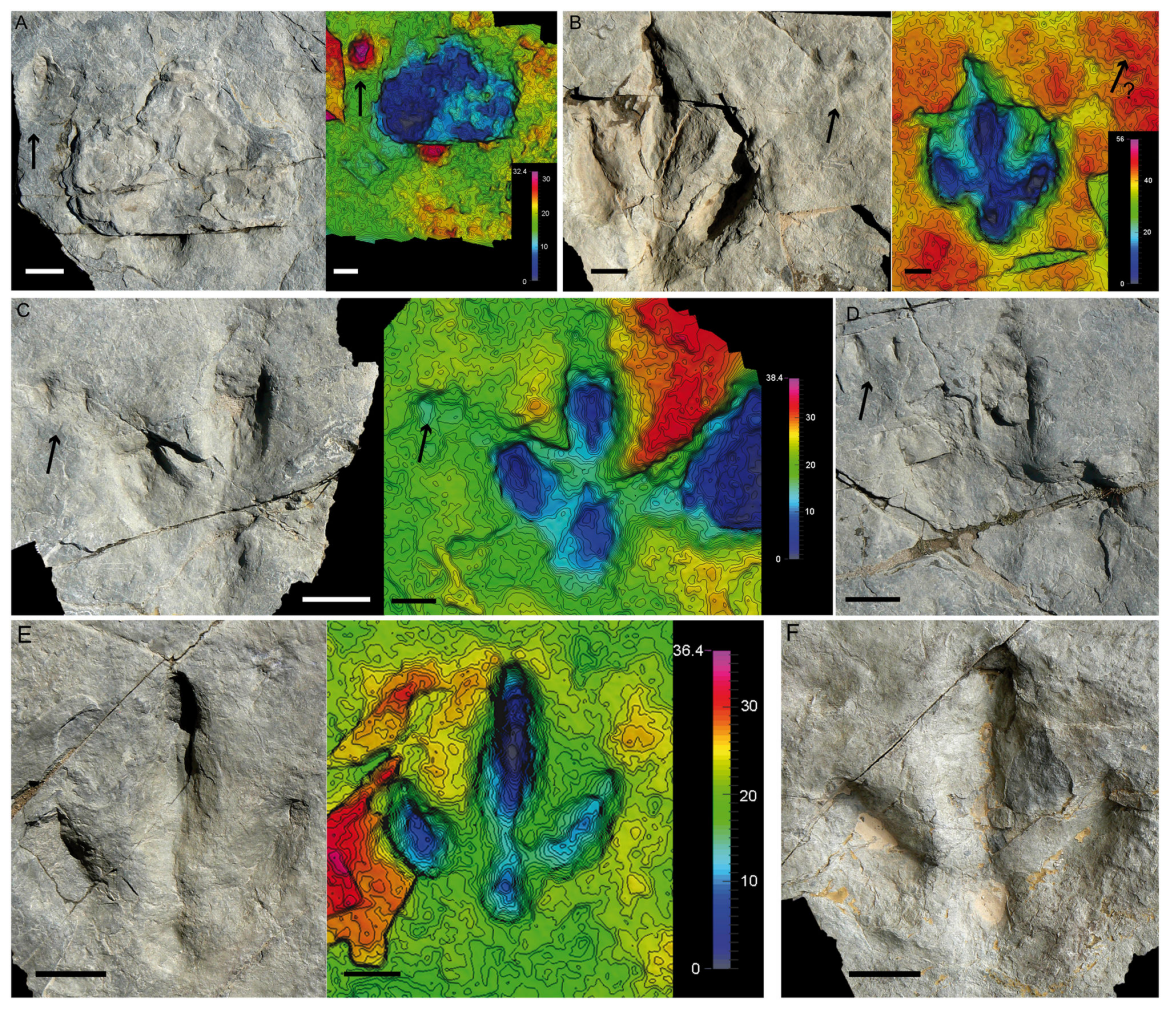
I?oncalensis tracks from La Peña tracksite.

2006 *Therangospodus oncalensis* Moratalla, 1993; emend. Lockley, Meyer and Moratalla, 2000 [16], figs. 2-6

2008 *Therangospodus oncalensis* [6], fig. 3 (TE-4)

#### Material.

See Materials and Methods.

#### Description

Medium-Sized (about 20-30 cm) tridactyl tracks, slightly longer than wide (length/width ratio about 1-1.2). They are characterized by a quadrangular to rounded heel pad impression with a lateral and medial notch, the track being reasonably symmetrical. The digits are robust, digit III being the longest and digits II and IV almost equal in length. The hypexes are also fairly symmetrical. There seem to be no discrete phalangeal pads, but some tracks show constrictions in the digits, so this absence could be a preservation bias. In the same way there is no evidence of sharp claw marks, though some tracks apparently evidence blunt claw marks. Interdigital angle II-IV generally varies from about 65° to 80°, interdigital angle II-III being slightly greater than interdigital angle III-IV. The trackway width is about 30-40 cm, which is rather narrow. The tracks show a slightly negative rotation of the digit III long axis from the trackway axis. The average step length is about 2.5 times the footprint length. Some trackways show possible manus tracks located laterally, close to digit IV.

#### Remarks

*Therangospodus oncalensis* clearly differs in some features with respect to the diagnosis of *Therangospodus*, especially in not being an “elongate and asymmetric theropod track” [15] though it shares “the coalesced, elongate oval digital pads” [15] and the apparent absence of discrete phalangeal pads. The trackway width and the “little or no rotation of the digit III long-axis from the trackway axis” [15] are also shared features. Nonetheless, the ichnospecies *T. oncalensis* clearly differs from *T. pandemicus* in having a smaller length/width ratio, a shorter pace length, a symmetrical and rounded to quadrangular “heel” pad impression and in the robustness and shorter length of the digits. Despite these differences, as suggested by Lockley et al. [15], “it is difficult to compare (the two ichnospecies) because the two samples originate from different continents and are samples of different sizes”. Moreover, they have a slightly different age (Late Jurassic vs. Early Cretaceous). The new data (see below) suggest that *T. oncalensis* should be removed from the ichnogenus and placed in a different ichnotaxon of ornithopod affinity. Within the ornithopod ichnotaxa, *T. oncalensis* shares features with tracks assigned to *Iguanodontipus*, a typical ichnotaxon from the Lower Cretaceous of Europe. Even so, given the current data some questions relating to the diagnosis of *Iguanodontipus*, its possible ontogenetic states and shared features with other tracks from the Jurassic-Cretaceous transition remain uncertain (see discussion). This prevents us from assigning the tracks to *Iguanodontipus*, and we have thus classified them as *I? oncalensis* using the open nomenclature due to the aforementioned doubts and in the absence of suitable comparison with the type material of *Iguanodontipus*.

### New data on the morphology and gait of *I? oncalensis*

#### The new tracks from Fuentesalvo: FTS18-FTS20

The new tracks (Figure 3) are located in the southwestern part of the tracksite, close to the ravine. There are at least three trackways with a similar direction to those reported by Barco et al. [16], so they probably belong to the same group, which is thus larger (at least 14 individuals). There seems to be a relationship among the tracks, although distinguishing the trackways is a difficult task (Figure 3). Of the new tracks, track FTS19 (Figure 4A-4B) is especially interesting on account of its preservation, while the others are not so well preserved. Within this track three microlayers of no more than one cm each can be observed. These microlayers have been observed in other described tracks (e.g., FTS7.5, FTS8.5, FTS10.3, FTS10.6, FTS11.3 and FTS12.2, Figure 4E). They are of particular interest because of the light they shed on the position of the original track-bearing surface. It is noteworthy that the microlayers can only be discerned inside some tracks (not in the rest of the tracksite), especially in the southwestern part of the tracksite. There are two possible explanations for the presence of these microlayers: 1- these layers represent the original tracking surface (or were close to it) and have been preserved only in some parts of the tracksite by the pressure of the dinosaurs when they passed, whereas in the other parts (without this pressure) these microlayers have been easily eroded. 2- The microlayers represent the overtracks, i.e., the layers that were deposited after the passing of the dinosaurs. In weighing up the two options, the first seems to be more parsimonious because the layers intrude into the substrate, so they were deformed by the passing of the dinosaurs. Moreover, it is difficult to explain the bias in the erosion of the microlayers and the absence of these layers in the footprint wall by the overtrack hypothesis. If our reasoning is correct, then this has important consequences for the preservation (Figure 4C-4D) of the holotype trackway (FTS1.3) of *I? oncalensis* and many other tracks in the tracksite, as this evidence would suggest that they are preserved as shallow undertracks (sensu Lockley [3]). Some parameters (FL, FW, FL/FW, II^IV, see Appendix S1) of FTS19 are not very different from those reported by Barco et al. [16]. Nonetheless, FTS19 has preserved some characters that cannot be seen in other tracks of the tracksite, especially in the morphology of the heel pad impression (quadrangular vs. rounded). Assuming that the holotype of *I? oncalensis* has been preserved as a shallow undertrack, the intriguing question is whether this quadrangular heel is the real morphology and it changes to round with depth (undertracks), as has been demonstrated by experimental ichnology to occur with some parameters [42-45]. Other parameters that are worthy of mention are the length/width ratio (1.1) and the AT (0.35) because both of these fall within the values for ornithopod tracks (see 8). For the tracks reported by Barco et al. [16], these parameters also lie within the range for ornithopods (see Appendix S2), varying in the FL/FW ratio from 1-1.2 and exceptionally 1.4 in FTS5 and FTS8, which are by far the worst-preserved tracks, and in the AT from 0.39 in the holotype (FTS1.3) to 0.46 (FTS4.4). 

#### La Peña tracksite

In the case of the La Peña tracksite, Aguirrezabala and Viera [35] and Moratalla [14] described part of trackway LP1 (Figure 5), which the latter author assigned to *cf. Therangospodus*. It is significant that the former authors consider *Iguanodon* as the candidate trackmaker for the trackway. Cleaning works have exposed eleven new tracks belonging to this trackway and two new trackways, LP2 and LP3, with nine and ten tracks respectively (Figure 5). As is the case with Fuentesalvo, the La Peña tracksite is composed of different layers in which the tracks are preserved. Analysis of track LP2.3 (Figure 6A) is particularly interesting because this track still preserves part of the cast inside the pes, so all the layers related to the tracks are well exposed. Furthermore, it also preserves an oval depression located laterally, close to digit IV of the pes, which might represent the manus track. The photogrammetric model enhances the presence and morphology of the manus impression (Figure 6A). 

The dotted lines indicate the tracks shown in figure 6.

A) Picture and photogrammetric 3D depth analysis model of track LP2.3. Note the preservation of the left pes track with the natural cast inside the footprint, and located laterally an oval depression in the possible tracking surface and interpreted here as a probable manus track. B) Picture and photogrammetric 3D depth analysis model of track LP2.12. C) Picture and photogrammetric 3D depth analysis model of track LP1.5. D) Picture of track LP1.3. Note in these three tracks the marks located laterally, in a similar position to that of the manus track in LP2.3. E) Picture and photogrammetric 3D depth analysis model of track LP3.8. F) Picture of track LP3.2. Note that the tracks from trackway LP3 do not show impressions of manus marks. Scale depth in the model in mm. Scale bar = 5 cm. 

Analysis of the layers in the rest of the tracksite suggests that the great majority are shallow undertracks. Nonetheless, despite being undertracks some probable manus tracks can be discerned close to the pes tracks (LP1.3, LP1.5, LP1.8, LP1.10, LP1.3, LP2.10 and LP2.12). In these cases, the morphology of the manus is not oval like track LP2.3 but consists of two or three smaller, individual oval/elongate marks (Figure 6B-6D). The photogrammetric models in LP1.5 and LP2.12 do not enhance the impressions as in manus track 2.3. It is noteworthy that in trackway LP3 it is very difficult to discern any sign of manus tracks in the field, even in the photogrammetric models (Figure 6E-6F). This bias in the presence/absence of manus prints is difficult to interpret. It might be a consequence of the tracks being undertracks, a consequence of overprinting or the result of it being a different gait (see below).

Analysis of the individual morphology of the tracks and the morphometric parameters suggests that these tracks share many features with the tracks at Fuentesalvo, such as the FL/FW ratio, the length of the digits (though the data from Barco et al. [16] were measured with a different methodology), pace length and stride length. The main differences between the various tracks from the two tracksites are the interdigital angles (see Appendix S1 and Table 1 in [16]). It is also worth underscoring that the tracks from La Peña show a rounded (not quadrangular) heel.

#### Undescribed tracks and trackways from other tracksites: Salgar de Sillas and Valloria IV:

**Figure 7:**
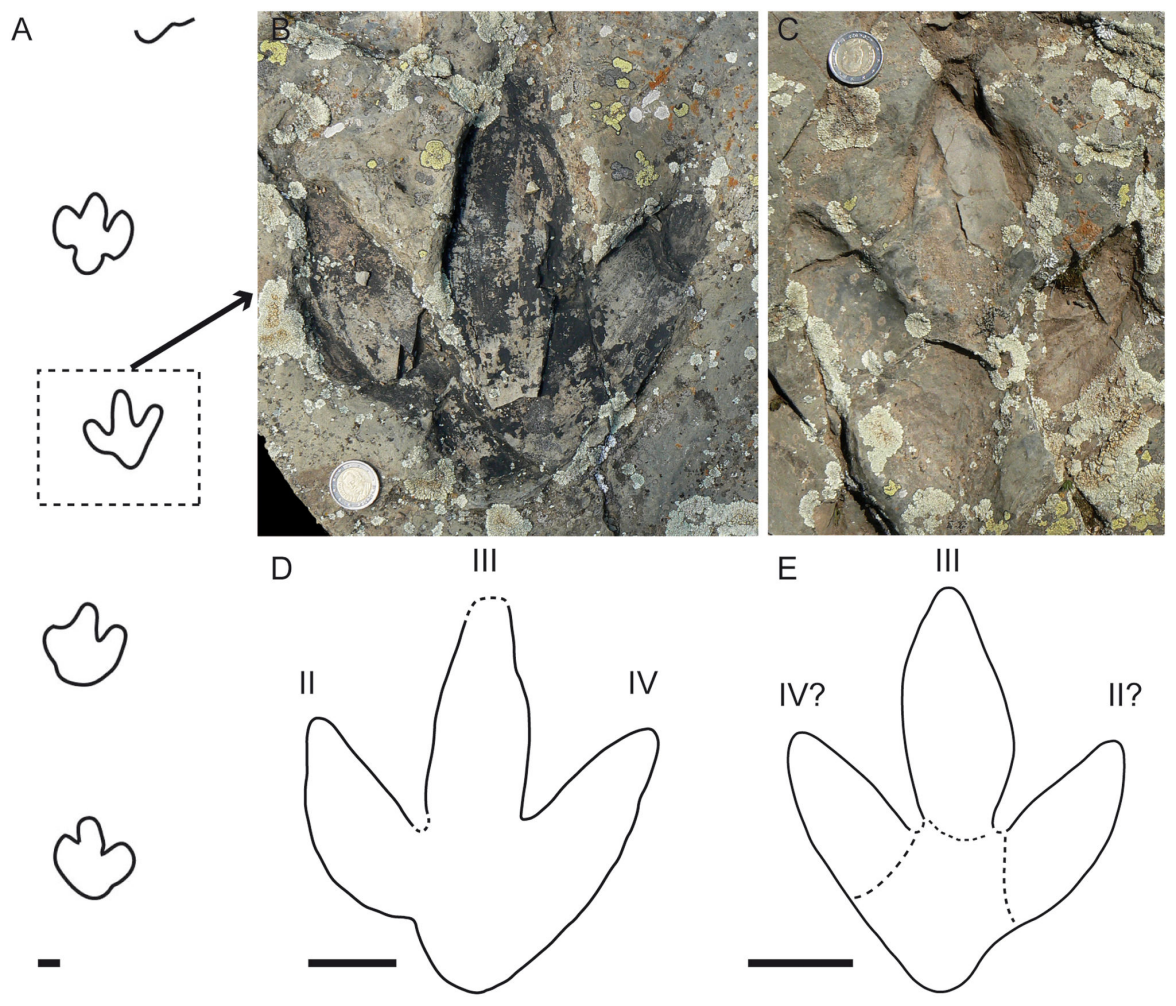
*I?oncalensis* tracks from the Salgar de Sillas tracksite. A) Sketch of trackway SS26. B) Picture of track SS26.3 (the tracks has been coloured in black with earthy tones). C) Picture of track SS27. D) Outline drawing of track SS26.3. E) Outline drawing of track SS27. Scale bar = 5 cm.

In both tracksites there is at least one trackway that has a similar morphology to the tracks from La Peña and Fuentesalvo and that has not been described. In Salgar de Sillas (Figure 7), trackway SS26 is composed of five tracks of no more than 25 cm in length, which resemble those of *I?oncalensis* both in morphology and other ichnological parameters (Appendix S1). Furthermore, a better-preserved isolated track (SS27) near the trackway is similar as well. In these tracksites there is no evidence of manus tracks. The other parameters (FL/FW ratio and AT) that suggest an ornithopod origin lie within the range of the other tracksites (1.2 and 0.39 in SS26, 1.24 and 0.45 in SS27).

**Figure 8:**
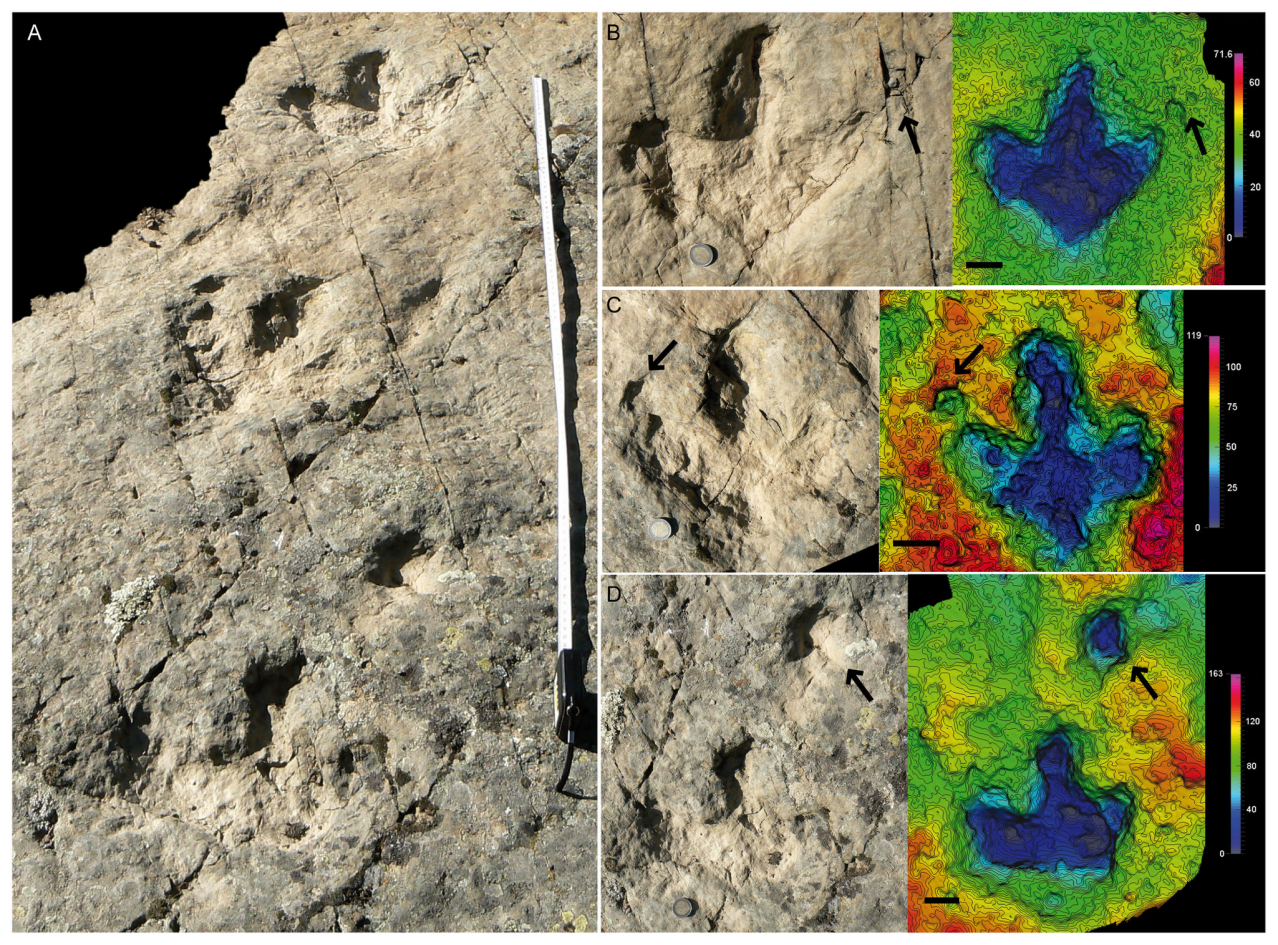
*I?oncalensis* trackway from the Valloria IV tracksite. A) Picture of the whole trackway VAIV1. B) Picture and photogrammetric 3D depth analysis model of track VAIV1.3. C) Picture and photogrammetric 3D depth analysis model of track VAIV1.2. D) Picture and photogrammetric 3D depth analysis model of track VAIV1.1. Scale depth in the model in mm. Scale bar = 5 cm.

In the case of *Valloria IV* a new trackway composed of at least three tracks has been recorded (Figure 8). Even though the tracks do not preserve such a clear morphology as the other tracksites, this trackway is of special interest because the tracks show an oval depression in the place where the manus track would be expected to be found (Figure 8). In the case of VAIV1.1 and VAIV1.3 there are certain doubts with respect to its location and preservation. In the former case (Figure 8D), the oval depression is located further away than in La Peña, while in VAIV1.3 there are some fractures that do not allow us to interpret the depression with certainty. The photogrammetric model of track VAIV1.1 does show an oval depression with a similar depth to that of the track. VAIV1.2 shows an oval depression in a place similar to the tracks at the La Peña tracksite, and this is also enhanced in the photogrammetric model. The parameters are also similar to those of the other tracksites (Appendix S1). The FL/FW ratio (1.01) and AT (0.44) also fall within the range of the *I? oncalensis* values. 

Other described tracksites associated with *I?oncalensis*: Los Tormos (LTR), Fuente Lacorte (FC), Valdecantos (VDC), Valdelavilla (VDV), La Losa II (LLII) and MNS-2002-96-2

**Figure 9:**
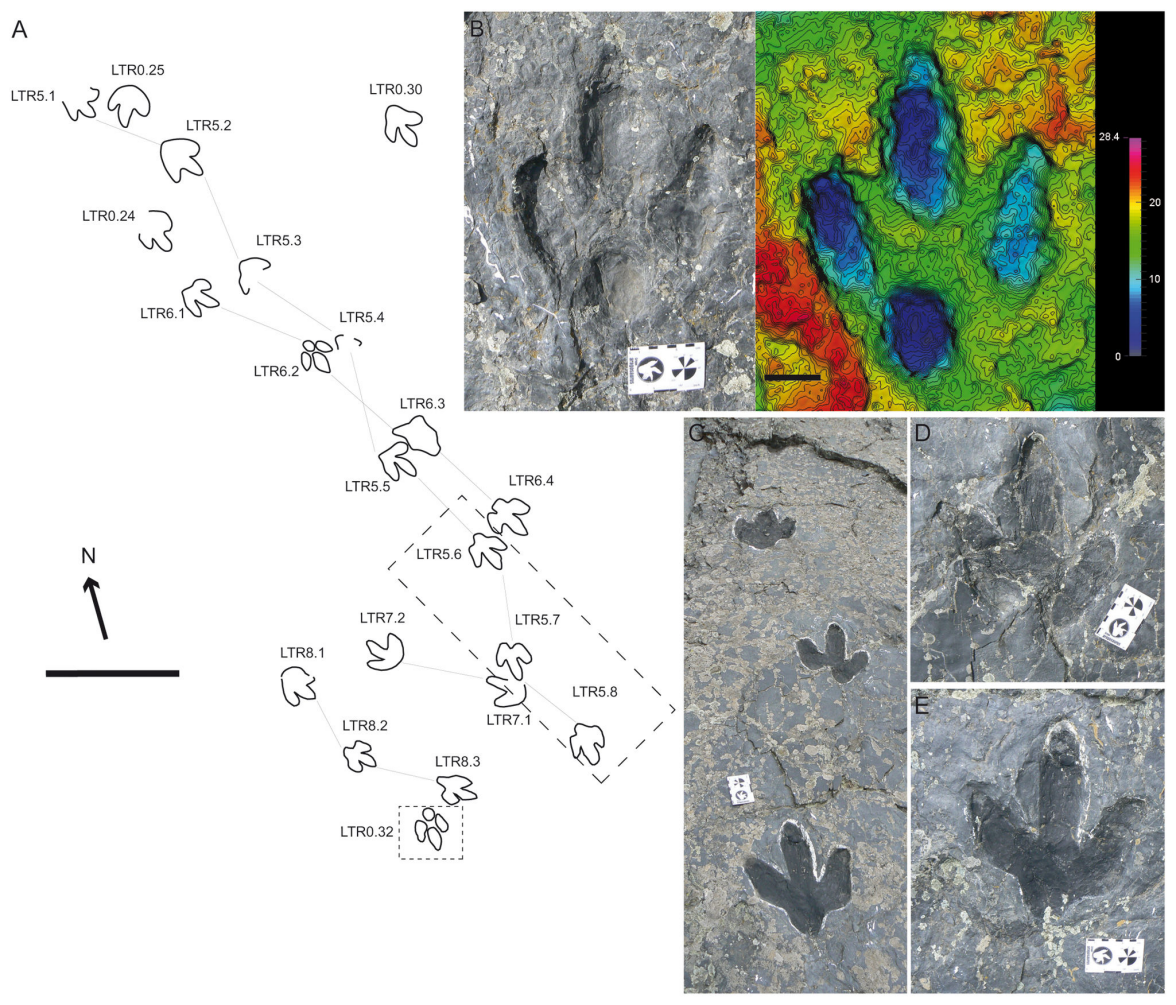
*I?oncalensis* tracks in the Los Tormos tracksite. A) Sketch map of the Los Tormos tracksite. B) Picture and photogrammetric 3D depth analysis model of the best-preserved track LTR0.32. C) Picture of part of trackway LTR5. D-E) Pictures of tracks LTR5.6 and LTR5.7. Note in LTR5.7 the two different layers where the track is impressed. Scale depth in the model in mm. Scale bar = 50 cm (A) and 5 cm (B). Scale (card) = 8 cm.

Moratalla [14] also cited the presence of *T. oncalensis* at the tracksites of Los Tormos, Fuente Lacorte and Valdecantos on the basis of a previous paper published by Aguirrezabala and Viera [36]. In the case of Los Tormos, Moratalla [14] suggests that tracks previously assigned to *Iguanodon* by Aguirrezabala and Viera [36] in fact correspond to *Therangospodus*. The cartography of level 2 shows that there are at least four trackways (Figure 9) and other isolated tracks that share the relevant general features. The preservation of some of them is not very good, as a consequence of erosion and because many of them are undertracks. The most interesting tracks correspond to those of trackway LTR5 (Figure 9C, 9D, 9E) and the isolated track LTR0.32 (Figure 9B) (assigned by Aguirrezabala and Viera [36] to a theropod, see Figure 7, page 8). The FL/FW ratio is about 1.16 in LTR5, while it is 1.18-1.32 in LTR0.32 (a value of 1.32 takes into account the probable claw mark). The AT is about 0.44 (Appendix S1). The other parameters are also within the range of *I? oncalensis*. It is noteworthy that in the case of track LTR5.7 some layers inside the track can be discerned. This provides crucial evidence that the great majority of the tracks from this level are undertracks. The photogrammetric model of track LTR0.32 enhances the pad in each digit.

In the case of Valdecantos, Moratalla [14] cited the presence of two trackways belonging to *Therangospodus*. During our revision of the tracksite, we have identified one trackway and other isolated tracks. The erosion that has taken place over more than 20 years prevents us from accurately comparing these tracks with those reported in the other tracksites. Nonetheless, at least one track still shows the rounded heel, typical of *I?oncalensis*. 

**Figure 10:**
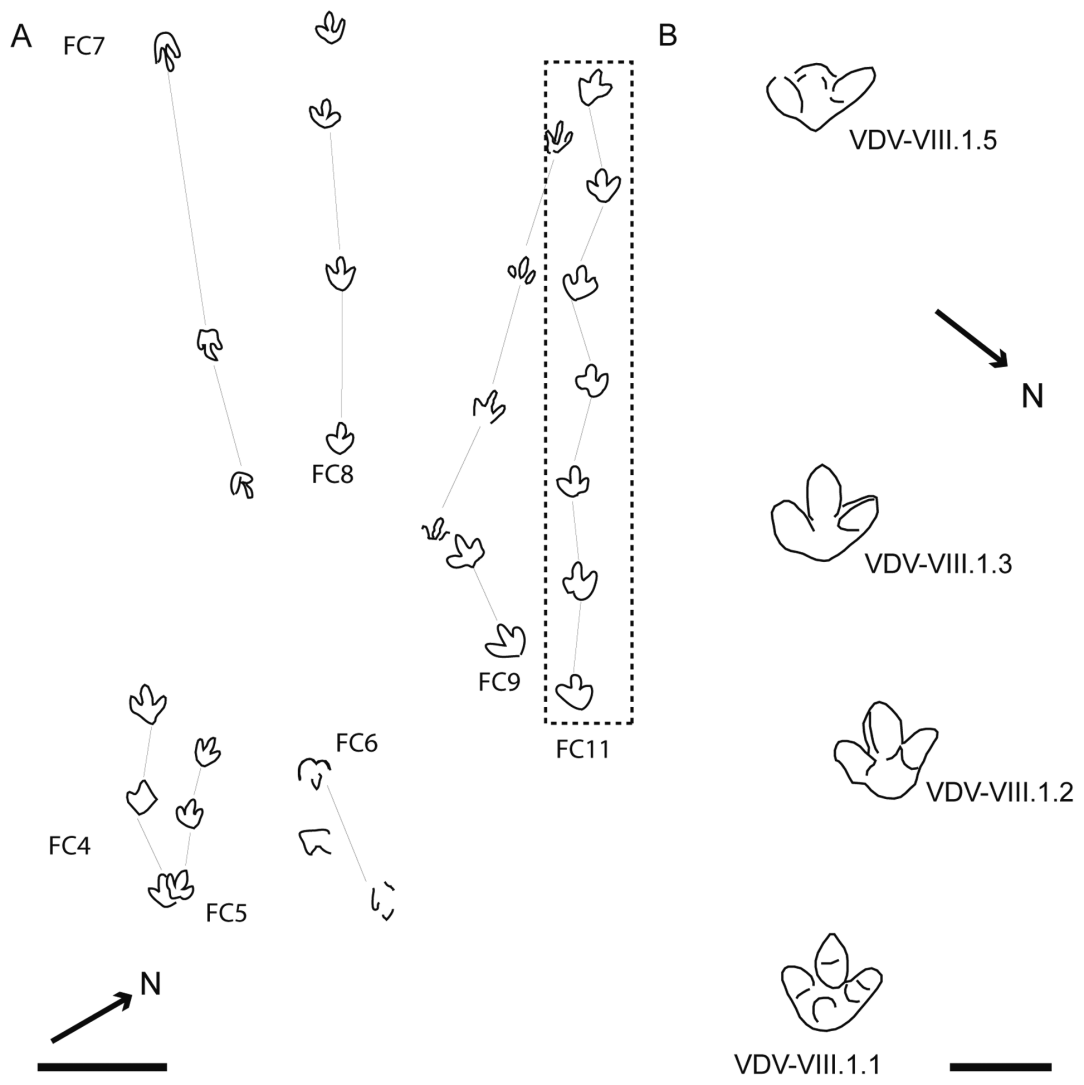
*I?oncalensis* tracks in other tracksites **I**. A) Sketch map of the Fuente Lacorte (level 10) tracksite. B) Sketch of the *I?oncalensis* trackway from the Valdelavilla (level VIII) tracksite (redrawn from [37]). Scale bar = 20 cm.

In Fuente Lacorte, Moratalla [14] also cited the presence of *Therangospodus*. The cartography of level 10 shows at least five trackways (Figure 10) that could resemble the general morphology of *I?oncalensis*. Nevertheless, the current preservation of the tracks is very poor. Only trackway FC11 (Figure 10A) has been included in our analysis. The FL/FW ratio is about 1 and the AT about 0.4-0.45. 

**Figure 11:**
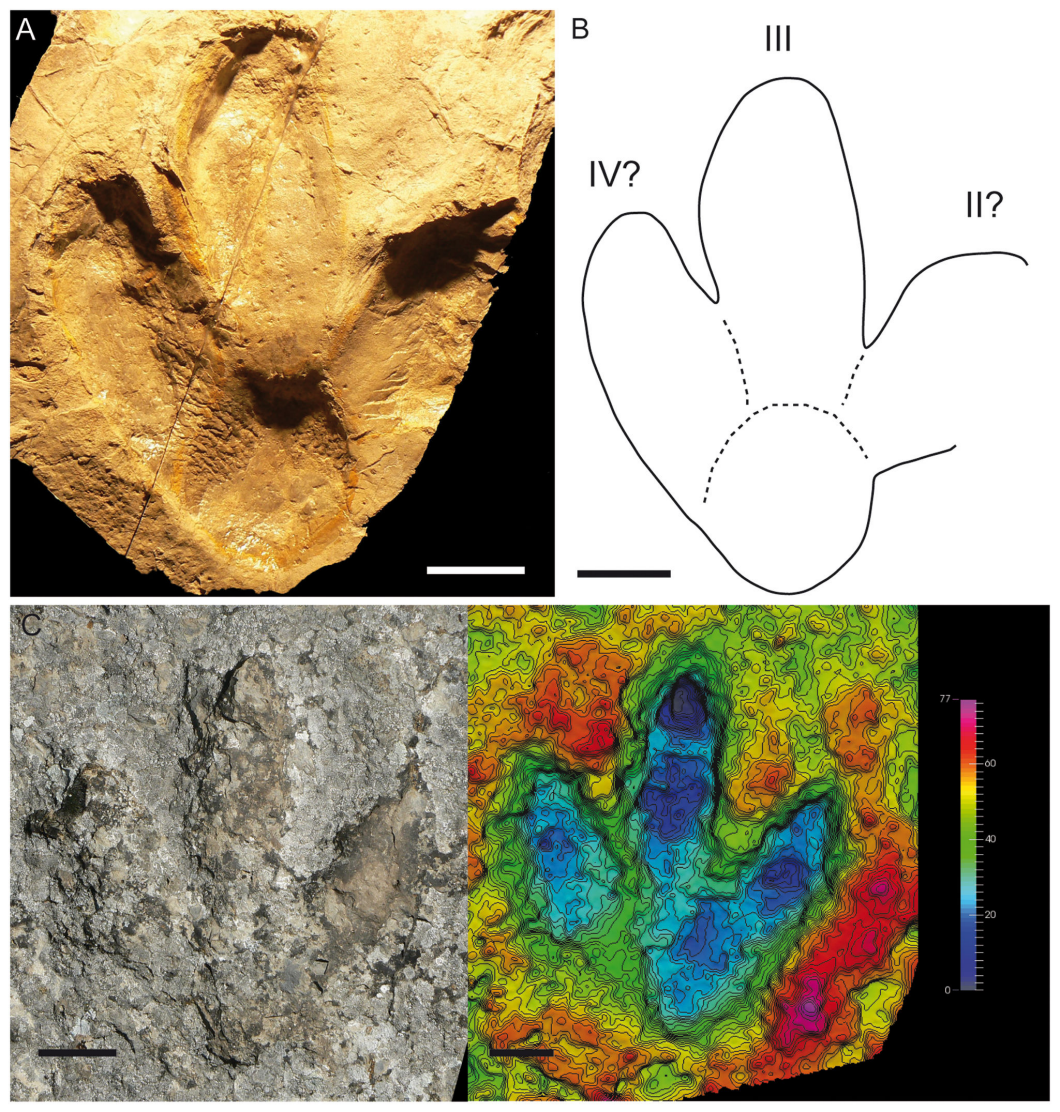
*I?oncalensis* tracks in other tracksites II. **A**) Picture of track MNS-2002-96-2 stored in the Museo Numantino (Soria). B) Outline drawing of track MNS-2002-96-2. C) Picture and photogrammetric 3D depth analysis model of track LLII1.4. Scale bar = 5 cm.

Furthermore, Hernández-Medrano et al. [6] cited the presence of *Therangospodus* in Valdelavilla (level VIII), referring to a trackway (Figure 10B) previously described by Pascual and Sanz [37]. The FL/FW ratio is 1.12 and the AT is about 0.42. *MNS-2002-96-2* (Figure 11A, 11B) is an isolated track from the collection of the Museo Numantino de Soria, which was figured by Fuentes-Vidarte et al. [39]. This also displays similar features to the other described tracks. The FL/FW ratio and AT are likewise comparable (1.16 and 0.42). A trackway figured by Fuentes Vidarte et al. [38] at the La Losa II tracksite also has similar features (Figure 11C). The FL/FW ratio is about 1.1 and the AT about 0.38. It is significant that the photogrammetric model enhances a depth difference in the proximal part of digit II and the heel impression (Figure 11C).

## Discussion

**Figure 12:**
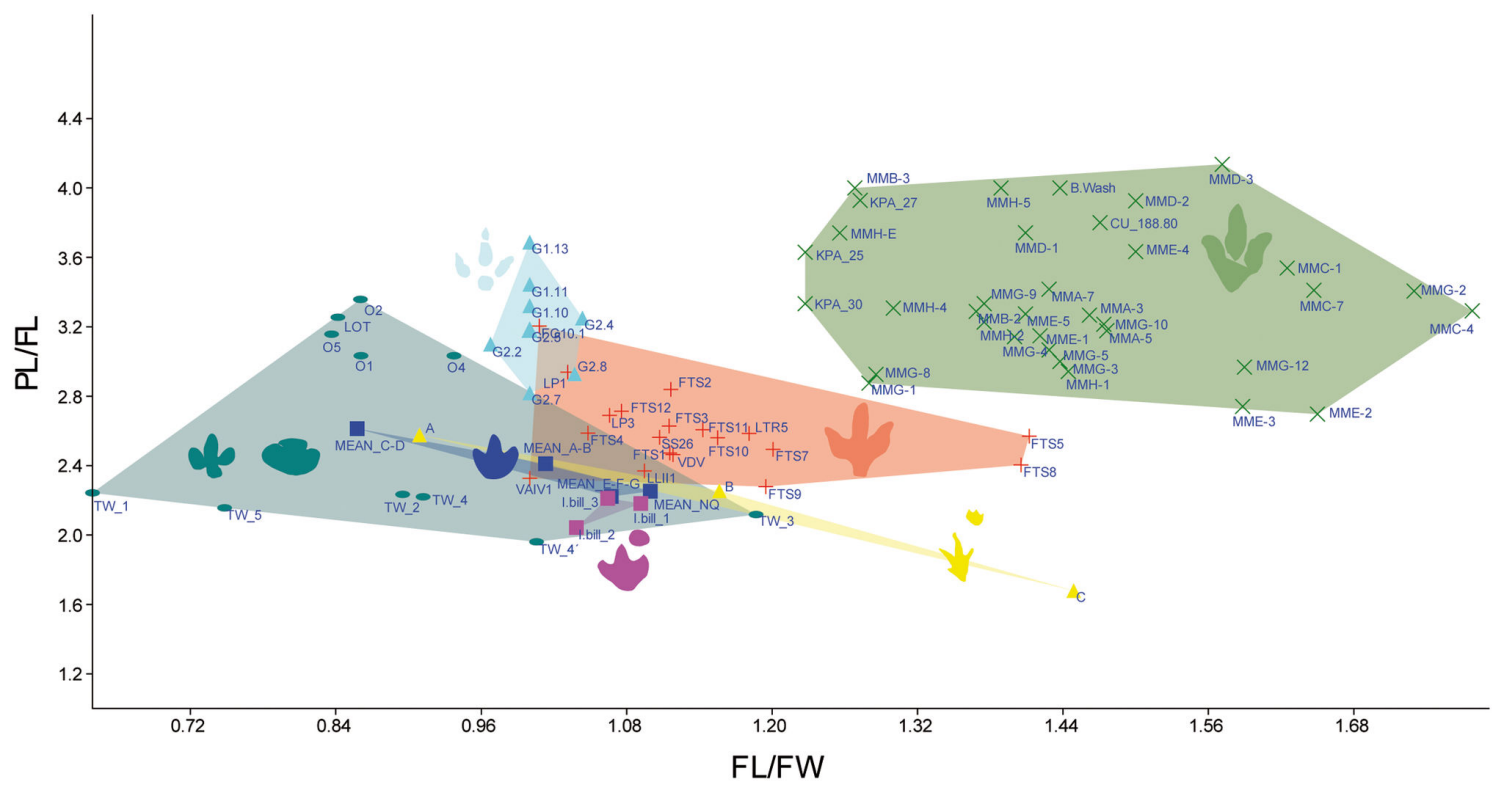
Bivariate analysis of the FL/FW ratio and PL/FL ratio of *I?oncalensis*, *T. pandemicus* and other ornithopod ichnotaxa. Plot showing the bivariate analysis results with FL/FW on the X axis and PL/FL on the Y axis. *T. pandemicus* (green), *I? oncalensis* (red), *Dineichnus* (light blue), *Iguanodontipus billsarjeanti* (pink), *Iguanodontipus burreyi* (dark blue), *Ornithopodichnus* (greenish blue), *Neoanomoepus* (yellow).

**Figure 13:**
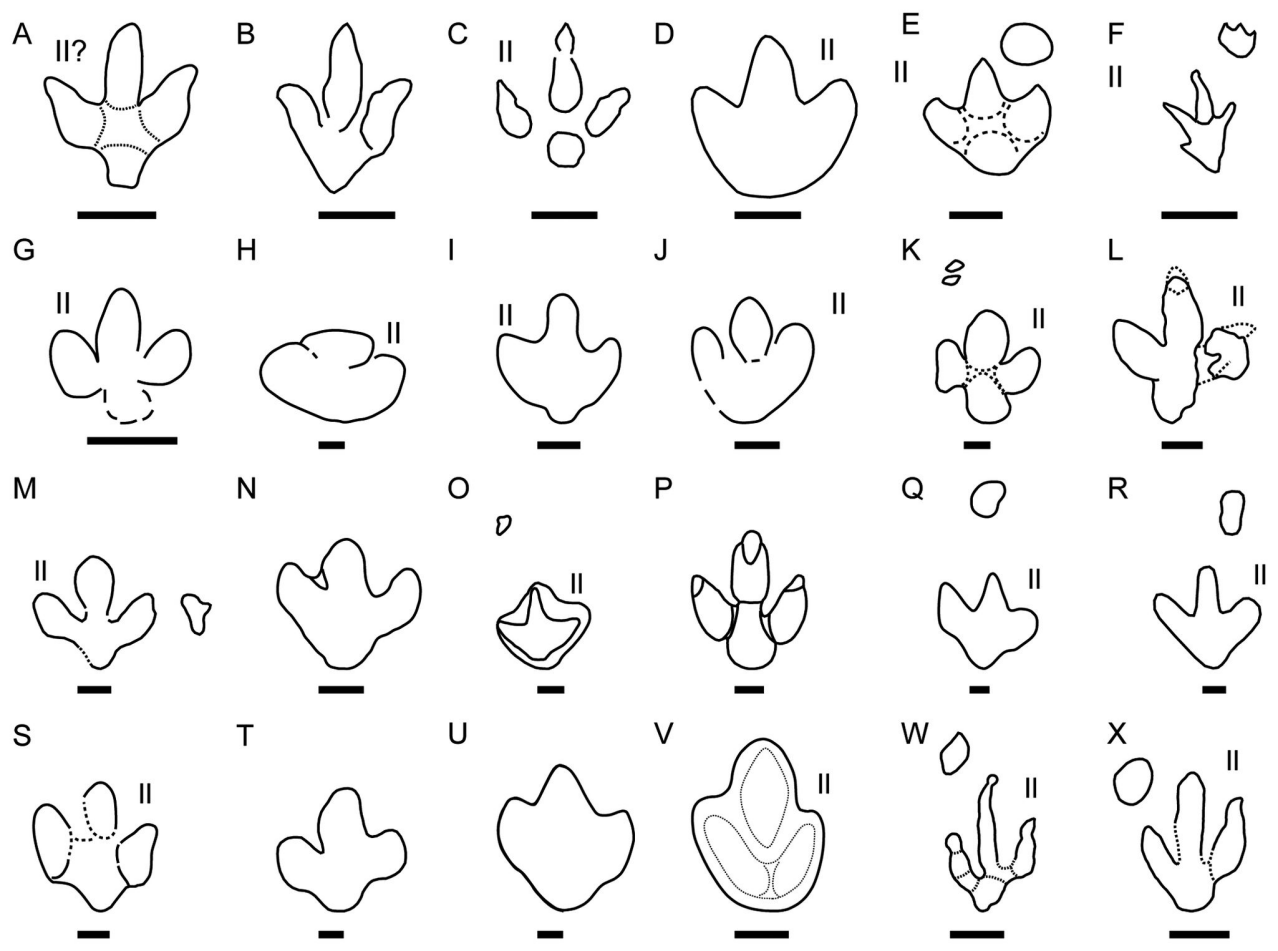
Comparison of *I? oncalensis*, *T. pandemicus* and other ornithopod ichnotaxa and ornithopod tracks. A) *I? oncalensis*; B) *T. pandemicus* from USA; C) *Dineichnus*; D) *Iguanodontipus burreyi*; E) *Iguanodontipus billsarjeanti*; F) *Neoanomoepus*; G-H) *Ornithopodichnus*; I-J) *Iguanodontipus* from the Las Cuestas I tracksite; K-M) *Iguanodontipus* from the Berriasian of Germany; N-P) *Iguanodontipus* from the Berriasian of the UK; Q-R) *Iguanodontipus* tracks from the Barremian of the Iberian Range (La Rioja and Burgos province). S) *Iguanondontipus* tracks from the Barremian of Portugal; T-U) *Iguanodontipus* tracks from the Barremian of the Iberian Range (Teruel province); V) Morphotype II described by Marty [24], from the Late Jurassic of Switzerland; W-X) ornithopod tracks from the Las Cerradicas tracksite. Redrawn from [8, 12, 13, 15, 24, 47-51, 53-55, 57-59, 61-66]. Scale bar = 10 cm.

Since the earliest publications [14–16,35,36,38,39] some of the tracks under consideration here have been considered theropodan in origin, while others have been considered ornithopodan. According to the analysis (Appendix S1, Appendix S2) we have undertaken at the different tracksites, the variations in these parameters fall within limits that indicate that all the studied tracks and trackways belong to the same ichnospecies. The new data obtained for *I?oncalensis* suggest that the trackmaker of this ichnospecies was an ornithopod instead of a theropod. This conclusion is reached on the basis of the length/width ratio, the AT, the short steps, a round/quadrangular heel impression (in the best-preserved trackways) and the presence of probable manus prints in some of the trackways. Comparing the FL/FW ratio and the PL/FL ratio in a bivariate analysis (Figure 12) with some of the typical small- to medium-sized (less than 45 cm, sensu [46]) ornithopod ichnotaxa (Figure 13 A-H) such as *Dineichnus* [47], *Iguanodontipus burreyi* [48], *Iguanodontipus billsarjeanti* [49], *Ornithopodichnus* [50,51], *Neoanomoepus* [10] and with the type ichnospecies of *Therangospodus pandemicus* [15], it can be observed that the data for *I?oncalensis* and the other tracksites from the Huérteles Formation are closer to the ornithopod ichnotaxa than to the theropod tracks assigned to *T. pandemicus*. The only two trackways (FTS5 and FTS8) where the FL/FW ratio is near the cluster of the sample from *T. pandemicus* are those that are poorly preserved, so we consider that these data probably do not represent the real morphology of the trackmaker. The FL/FW ratio is generally below the threshold value of 1.25 which Moratalla [1] considered to discriminate between theropod and ornithopod tracks.

**Figure 14:**
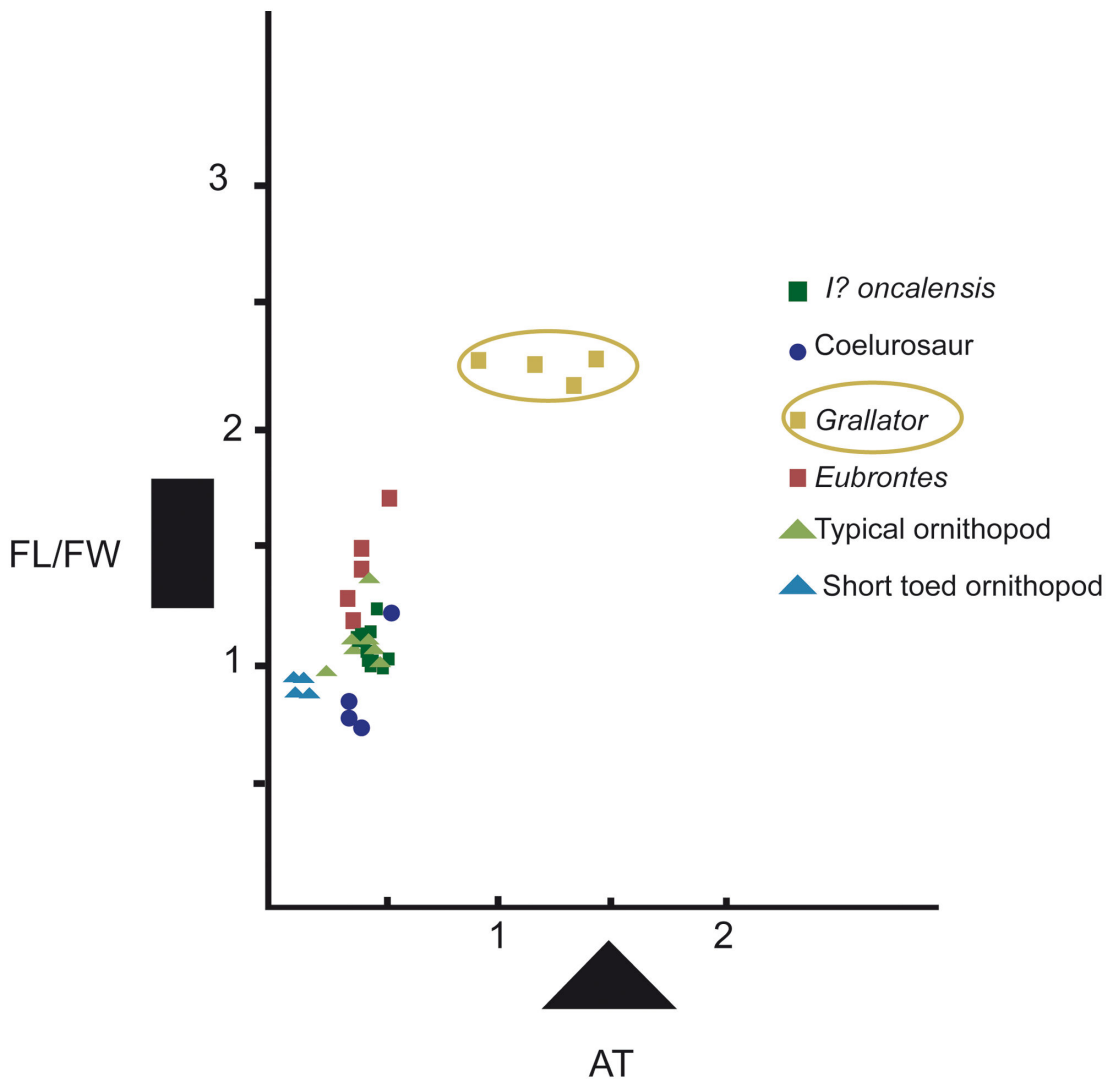
Bivariate analysis of the FL/FW ratio vs. **AT **(anterior triangle length-width ratio) **of** ***I?oncalensis*** and **other** **tridactyl **(theropod and ornithopod) **ichnotaxa** (after [8]).

Comparing the AT values in the same way, these vary from 0.35 to 0.46 for the tracks under study (Appendix S2; Figure 14). These values are quite similar to those reported by Lockley [8] for *Dineichnus* (0.45-0.51, Figure 13C), *Iguanodontipus* (0.39, Figure 13D) and *Neoanomoepus* (0.48, Figure 13F) and mainly fall within the range that the author considers typical of ornithopods. Furthermore, the quadrangular to rounded heel pad impression is a feature that has not been described in other theropod ichnotaxa (see 8,15,52).

Tracks belonging to *I?oncalensis* have marked features of ornithopod tracks such as the length/width ratio, the AT, the short pace length, and manus impressions in some of the trackways. These data suggest that the ichnospecies *oncalensis* (Figure 13A) should be removed from the ichnogenus *Therangospodus*, typical of theropod tracks (Figure 13B), and placed in an ornithopod ichnotaxon. The typical ornithopod ichnotaxa described in the Late Jurassic and Early Cretaceous of Europe are *Dineichnus* (Figure 13C) and *Iguanodontipus* (Figure 13D; 13E). The former is characterized by small to medium tridactyl quadripartite symmetrical tracks, about as wide as long and with a distinctive circular heel pad impression [47]. It is significant that some *I?oncalensis* tracks seem to resemble this general morphology (Figures 3-11 and Figure 5 in [16]). The main differences between *I?oncalensis* and *Dineichnus* lie in the length/width ratio (which is slightly higher in *I?oncalensis*), the interdigital angles (which are lower in *I?oncalensis*) and the pace length (which is shorter in *I?oncalensis*).

**Figure 15:**
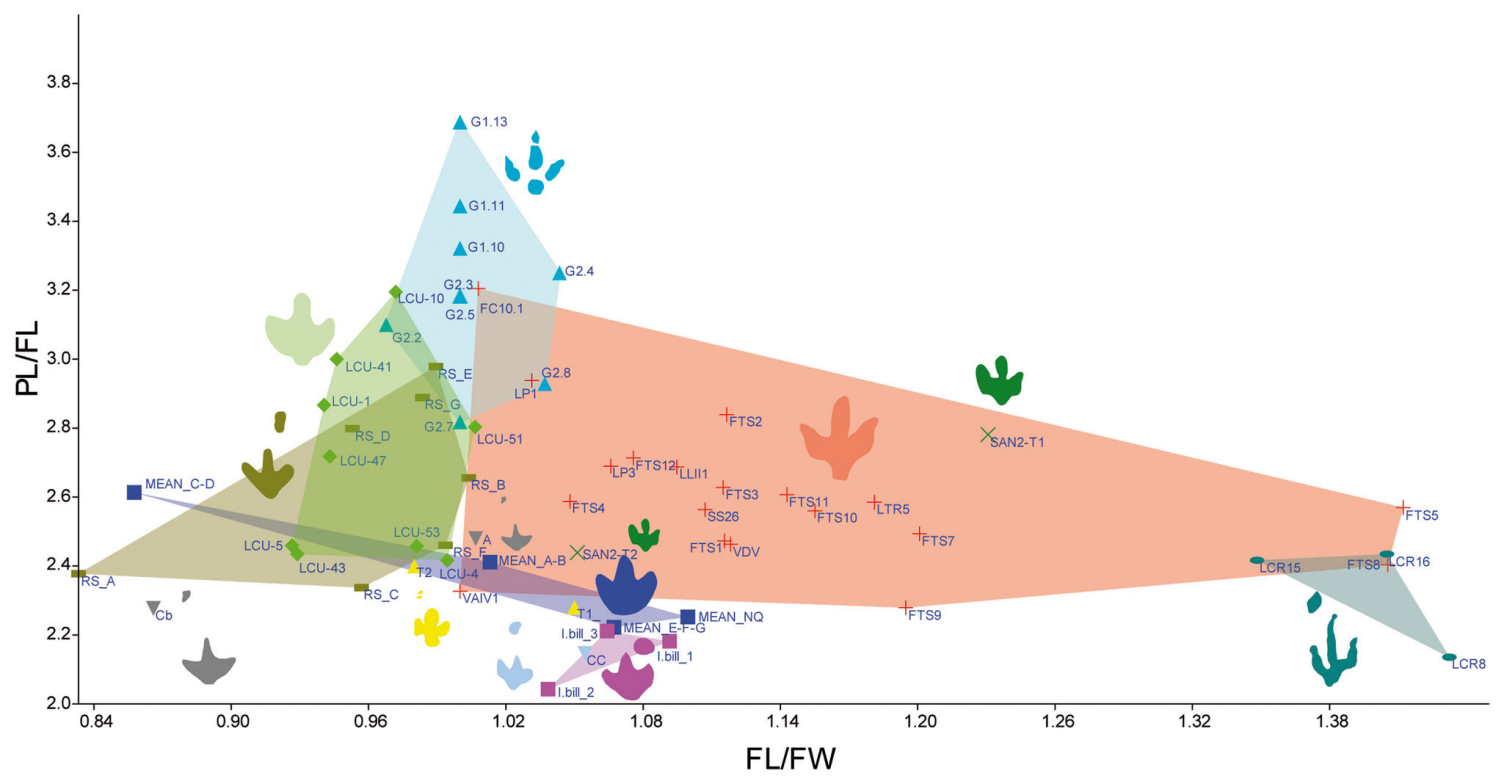
Bivariate analysis of the FL/FW ratio and PL/FL ratio of *I? oncalensis* and other ornithopod ichnotaxa and ornithopod tracks from the Late Jurassic-Early Cretaceous. Plot showing the bivariate analysis results with FL/FW on the X axis and PL/FL on the Y axis. *I?oncalensis* (red), *Iguanodontipus* *billsarjeanti* (pink), *Iguanodontipus* *burreyi* (dark blue), *Iguanodontipus* from the Huérteles Formation (light green), *Dineichnus* (light blue), *Iguanodontipus* from Praia Santa (green), *Iguanodontipus* *sp*. from Obernkirchen (yellow), *Iguanodontipus* from the Purbeck Limestone Group (grey), *Iguanodontipus* from Regumiel de la Sierra (dark green), *Iguanodontipus* from Cabezón de Cameros (greyish blue), unnamed tracks from Las Cerradicas (greenish blue).

As regards *Iguanodontipus*, two ichnospecies in the ichnogenus have been described: *Iguanodontipus burreyi* (Figure 13D) from the Berriasian of the UK [48], and *Iguanodontipus billsarjeanti* (Figure 13E) from the Late Aptian of Switzerland [49]. Other tracks assigned to *Iguanodontipus* have been described dating to the Berriasian in Spain (Figure 13I, 13J), likewise in the Huérteles Formation [53], and in Germany (Figure 13K-M, [12,54–56]). Other “iguanodontian” tracks (Figure 13N, 13O) have been described in the Purbeck Limestone Group (Berriasian) in England [57,58] and subsequently assigned to *Iguanodontipus* sp. [27]. Furthermore, *Iguanodontipus* tracks have also been described in other Early Cretaceous sites in the Wealden of England (Figure 13P, [59,60]) and in the Iberian Peninsula (Figures 13Q-U, [61-65]). As regards the diagnosis of the ichnogenus proposed by Sarjeant et al. [48], *I?oncalensis* does not meet the criteria of having “three digits of similar length” or “an equilateral triangle shaped digit III”, but it shares some features, such as digits II and IV with an “isosceles triangle shape” with rounded distal ends. The diagnosis of *Iguanodontipus* does not mention any features of the heel pad impression (quadrangular to round) and the posteromedial and posterolateral indentation in this part of the track, which in *I?oncalensis* is very characteristic. Nonetheless, similar features can be seen in the *Iguanodontipus* tracks described in Germany by Diedrich [55]. This author suggests (see Figure 6, page 221) that this variation may be a result of the preservation of the holotype material of *Iguanodontipus burreyi*. Generally, the tracks assigned to *Iguanodontipus* are slightly larger in size than *I?oncalensis* (see Appendix S3), with the great majority of the tracks being more than 25 cm in length. The presence of larger tracks assigned to *Iguanodontipus* in the Huérteles Formation could be taken to suggest that *I?oncalensis* should be placed in this ichnogenus. Some parameters such as the interdigital angles or pace angulation do not vary greatly between the two populations from the Huérteles Formation (means of 71° and 160°-165° respectively in the *Iguanodontipus* sample according to [53]). Nonetheless, the plot (Figure 15) showing the FL/FW ratio and PL/FW ratio reveals significant differences in these parameters between the two populations of ornithopods from the Huérteles Formation, though some of the tracks assigned to *Iguanodontipus* from other European tracksites [48,49,55,57,58,61-63] are close to the data for *I?oncalensis*. 

In a recent paper, Hornung et al. [12] reject the use of the ichnogenus *Iguanodontipus* “among the large ornithopod tracks from the LSB” (Lower Saxony Basin) and suggest that “this ichnogenus was introduced by Sarjeant et al. (1998) for a distinct morphotype of tridactyl ornithopod tracks and not intended as a catch-all ichnotaxon for Lower Cretaceous iguanodontian tracks”. The authors distinguish at least two or three morphotypes among the ornithopod tracks left by bipedal and quadrupedal individuals in various ontogenetic stages. This interpretation throws new light on the relationship between the *I?oncalensis* tracks and the *Iguanodontipus* tracks from the Huérteles Formation, and the question of whether the former might represent a juvenile ontogenetic state of the latter. Nonetheless, with the current data it is difficult to compare the two samples, considering the preservation state of the latter.

I*? oncalensis* also shares certain features with various unnamed tracks. It shares some features with morphotype II (Figure 13V) described by Marty [24] from the Late Jurassic of Switzerland, as was suggested by the author himself. The tracks share their medium size and robust digits, with digit III being the longest and II and IV of similar size. The fairly symmetrical hypexes and the extended heel are also shared features. In the case of morphotype II, the FL/FW ratio is greater (1.3-1.5). The author also suggests that this morphotype shares certain features with *Iguanodontipus*. Furthermore, as has been noted [13,26], the unnamed ornithopod tracks from Las Cerradicas (Figure 13W-X) also have features in common with some *Therangospodus* tracks. *I? oncalensis* shares the round “heel” pad impression, the similar length of digits II and IV, and the moderate interdigital divarication with the unnamed tracks from Las Cerradicas. The main differences reside in the morphology of the claw marks (apparently blunter in *I?oncalensis*), the FL/FW ratio (slightly lower in *I?oncalensis*), and in the degree of symmetry (*I?oncalensis* more symmetrical). As was suggested by Lockley [9], the tracks from Las Cerradicas are “intermediate between *Dineichnus* and *Iguanodontipus*”, so they could represent an undescribed ichnotaxon not formally erected. 

As we have seen in the course of this discussion, the current status of tracks assigned to medium-sized ornithopods from the Late Jurassic-Early Cretaceous interval is not well understood at all. The ichnotaxon *Iguanodontipus* includes medium to large European ornithopod tracks that range from the Berriasian to the Late Aptian in age. The characters that determine the ichnospecies and the ichnogenera within *Iguanodontipus* are not well defined at all, so despite the similarities between *I? oncalensis* and some of the tracks assigned to *Iguanodontipus*, we prefer to use the open nomenclature until further studies have been carried out. Given the current state of knowledge, it is difficult to determine whether *I?oncalensis* is a third ichnospecies within *Iguanodontipus* in its own right, whether it is just an ontogenetic variation of one of the existing ichnospecies, or whether it in fact represents an undescribed ichnogenus to which various tracks from the Late Jurassic and the first stages of the Cretaceous might correspond. 

### Preliminary Notes on the Gait of *I? oncalensis*

The data observed in the bivariate analysis show that the PL/FL ratio (Figure 15, Appendix S4) for *I? oncalensis* varies from 2.4 to 2.9, the highest value being trackway LP1, which is in fact one of those that preserves some evidence of manus tracks. These values are markedly lower than those from *T. pandemicus* (2.69-4) or *Dineichnus* (2.9-3.6), which are clearly bipedal ichnotaxa, and are closer to those from some *Iguanodontipus* tracks. The variation among the tracks assigned to the latter ichnotaxon is considerable. Values vary from 2.2-2.6 in *I. burreyi* (bipedal trackways); 2-2.2 in *I. billsarjeanti* (quadrupedal trackways); 2.4-2.7 in the *Iguanodontipus* sp. from Praia Santa (bipedal trackways); 2.28-2.4 in the *Iguanodontipus* sp. from Obernkirchen (quadrupedal trackways); 2.4-3.1 in the *Iguanodontipus* sp. from the Huérteles Formation (bipedal trackways); 2.2-2.4 in the *Iguanodontipus* sp. tracks from the Purbeck Limestone Group (quadrupedal trackways); 2.15 from a quadrupedal trackway [61] and 2.3-2.9 from bipedal trackways in Spain [62] subsequently assigned to *Iguanodontipus* [48]. 

 Among other ichnotaxa, the variation in the values of *Ornithopodichnus* tracks from different tracksites is notable, these values ranging from 3-3.2 in those reported by Lockley et al. [51] and 1.9-2.24 in those reported by Kim et al. [50]. In the former case, the trackways are clearly bipedal, while in the latter case poorly preserved manus traces have been described. In *Neoanomoepus* the values vary from 1.6-2.5, these being quadrupedal trackways. In other quadrupedal trackways, the values vary from 2.1-2.4 in the unnamed tracks from Las Cerradicas, while in large ornithopod tracks from the Dakota Group described in the literature the values are about 2.0-2.48 [66]. 

The variation among the *I?oncalensis* trackways lies within the range of the aforementioned tracks (Appendix S4). The values do not vary much between the apparently bipedal and quadrupedal trackways, although they are closer to the bipedal ornithopod trackways (*I. burreyi*, *Iguanodontipus* tracks from Praia Santa, the Huérteles Formation, and Regumiel de la Sierra, *Ornithopodichnus* “*1*”, with values ranging from 2.2-3.2) than to the quadrupedal ornithopod trackways (*I. billsarjeanti*, *Iguanodontipus* tracks from Obernkirchen, the Purbeck Limestone Group, Regumiel de la Sierra, Cabezón de Cameros, *Ornithopodichnus* “*2*”, *Neoanomoepus* and the unnamed tracks from Las Cerradicas, with values around 1.6-2.5). 

In analysing these data, it is difficult to interpret whether the gait of *I?oncalensis* is bipedal or quadrupedal because the values seem to be midway between the two. The bias against manus prints with respect to the tracking surface observed in the tracksites of Las Cerradicas [13] and La Peña raises the question of a possible bias in the tracksites where there is no apparent evidence of manus tracks. Moreover, another possible bias associated with the overprinting of the manus during locomotion should be borne in mind [3,67]. A third possible explanation could be that the dinosaurs, though facultatively quadrupedal, also moved in a bipedal way, so some trackways represent a bipedal gait while others represent the quadrupedal gait [62]. Nonetheless, the high values (2.3, 2.6, 2.9) shown by the trackways with evidence of manus tracks are closer to those of the bipedal trackways from the literature (Appendix S4), which lends little support to the third hypothesis. Furthermore, different gaits should produce great variation in these values as a result of the change in stance associated with different modes of locomotion, though such variations have not been shown among the bipedal and quadrupedal trackways from Regumiel de La Sierra [62].

To turn to ornithopod osteological remains (see 68, what seems clear is that during Berriasian times there is no evidence of derived ornithopods such as *Iguanodon*, so the trackmakers are probably ornithopods more basal than Hadrosauriformes. Moreover, the pedes [69] of these derived ornithopods are probably too robust and large to produce this kind of tracks. In a recent paper, Maidment and Barret [70] suggest that the acquisition of features associated with quadrupedal locomotion in non-hadrosaurid iguanodontids was variable and conclude that quadrupedalism correlates with derived forms (“more derived than *Equijubus*”). It is worth underscoring that these derived forms are younger forms than the candidate trackmakers of *I?oncalensis*. Thus, even though the evidence of manus tracks is poor it is highly interesting in that it adds new data to the debate on how some basal iguanodontids moved.

## Conclusions

This review of *Therangospodus oncalensis* material from the type tracksite and other tracksites from the same formation (Huérteles) suggests that the trackmaker was an ornithopod rather than a theropod. The characters that suggest its inclusion within Ornithopoda are the length/width ratio, a round to quadrangular heel pad impression, the probable presence of manus tracks in some of the trackways, the AT values and short steps. On the basis of these data, we have provisionally classified the material as *Iguanodontipus? oncalensis*, taking into account that even though the material is reasonably well-preserved it is not at all clear where it should be placed among the ornithopod ichnogenera. Nonetheless, it shares various features with tracks assigned to *Iguanodontipus* from different tracksites in Europe, mainly in the Berriasian of Germany and the UK. Our analysis of the PL/FL ratio sheds light on the gait of ornithopods and may prove an interesting tool for discriminating between possible quadrupedal and bipedal trackways. This reassessment reduces the number of theropod tracks (increasing the ornithopod tracks) in the Huérteles Formation, so the palaeoecological implications of its ichnodiversity may vary [6,7]. Furthermore, this reassessment also suggests that the gregarious behaviour described [16] in the Fuentesalvo tracksite took place among ornithopods (instead of theropods) and that the group was larger (at least three individuals or more). 

## Supporting Information

Appendix S1 **Table with the measurements taken in the *I?oncalensis* trackways in the Huérteles Formation. Abbreviations: see text in Materials and Methods.**
(XLS)

Appendix S2 **Table with the mean values of the parameters analysed (PL/FL ratio, FL/FW ratio and AT) in the *I?oncalensis* sample. Abbreviations: see text in Materials and Methods.**
(XLS)

Appendix S3 **PL/FL ratio and FL/FW ratio in the tridactyl ichnotaxa analysed.**
(XLS)

Appendix S4 **PL/FL ratio and gait of some of the tridactyl ichnotaxa and other ornithopod tracks analysed.**
(XLS)

## References

[1] Moratalla JJ, Sanz JL, Jiménez S (1988) Multivariate analysis on Lower Cretaceous dinosaur footprints: discrimination between ornithopods and theropods. Geobios 21 (4): 395-408. doi:10.1016/S0016-6995(88)80042-1.

[2] Thulborn T (1990) Dinosaur tracks. London: Chapman and Hall. 410 pp.

[3] Lockley MG (1991) Tracking dinosaurs: A new look at an ancient world. Cambridge: Cambridge University Press. 238 pp.

[4] Dalla Vecchia FM, Tarlao A (2000) New dinosaur track sites in the Albian (Early Cretaceous) of the Istrian peninsula (Croatia). Part II. Paleontology. Memorie di Scienze Geologiche 52 (2): 227-292.

[5] Romilio A, Salisbury SW (2011) A reassessment of large theropod dinosaur tracks from the mid-Cretaceous (late Albian-Cenomanian) Winton Formation of Lark Quarry, central-western Queensland, Australia: A case for mistaken identity. Cretaceous Research 32: 135-142. doi:10.1016/j.cretres.2010.11.003.

[6] Hernández Medrano N, Arribas Pascual, C, Latorre Macarrón P, Sanz Pérez E (2008) Contribución de los yacimientos de icnitas sorianos al registro general de Cameros. Zubía 23-24: 79-120.

[7] Moratalla JJ, Hernán J (2010) Probable palaeogeographic influences of the Lower Cretaceous Iberian rifting phase in the Eastern Cameros Basin (Spain) on dinosaur trackway orientations. Palaeogeography, Palaeoclimatology, Palaeoecology 295: 116-130. doi:10.1016/j.palaeo.2010.05.027.

[8] Lockley MG (2009) New perspectives on morphological variation in tridactyl footprints: clues to widespread convergence in developmental dynamics. Geological Quarterly 53 (4): 415-432.

[9] Lockley MG (2009) Some comparisons between dinosaur-dominated footprint assemblages in North America and Europe. In: Huerta P Torcida F Actas de las IV Jornadas Internacionales sobre Paleontología de Dinosaurios y su Entorno. Burgos: Salas de los Infantes. pp. 121-138.

[10] Lockley MG, McCrea RT, Matsukawa M (2009) Ichnological evidence for small quadrupedal ornithischians from the basal Cretaceous of SE Asia and North America: implications for a global radiation. In: Buffetaut E Cuny G Le Loeuff J Suteethorn V Late Palaeozoic and Mesozoic Ecosystems in SE Asia. The Geological Society, London, Special Publications 315: 255-269

[11] Cobos A (2011) Los dinosaurios de Teruel como recurso para el desarrollo territorial. PhD thesis, Universidad del País Vasco. 560 pp.

[12] Hornung JJ, Böhme A, van der Lubbe T, Reich M, Richter A (2012) Vertebrate tracksites in the Obernkirchen Sandstone (late Berriasian, Early Cretaceous) of northwest Germany - their stratigraphical, palaeogeographical, palaeoecological, and historical context. Paläontologische Zeitschrift 86: 231-267. doi:10.1007/s12542-012-0131-7.

[13] Castanera D, Vila B, Razzolini NL, Falkingham PL, Canudo JI et al. (2013) Manus Track Preservation Bias as a Key Factor for Assessing Trackmaker Identity and Quadrupedalism in Basal Ornithopods. PLOS_ONE 8(1): e54177. doi:10.1371/journal.pone.0054177. 23349817 PMC3551957

[14] Moratalla García JJ (1993) Restos indirectos de dinosaurios del registro español: Paleoicnología de la Cuenca de Cameros (Jurásico superior-Cretácico inferior) y Paleoología del Cretácico superior. PhD thesis; Universidad Complutense de Madrid 727 p.

[15] Lockley MG, Meyer CA, Moratalla JJ (1998) *Therangospodus*: trackway evidence for the widespread distribution of a Late Jurassic theropod with well-padded feet. GAIA. Aspects of Theropod Paleobiology 15: 339-353.

[16] Barco JL, Canudo JI, Ruiz-Omeñaca JI (2006) New data on *Therangospodus* *oncalensis* from the Berriasian Fuentesalvo tracksite (Villar del Río, Soria, Spain): an example of gregarious behaviour in theropod dinosaurs. Ichnos 13: 237-248. doi:10.1080/10420940600843682.

[17] Gierlinski GD (2009) A preliminary report on new dinosaur tracks in the Triassic, Jurassic and Cretaceous of Poland. In: Huerta P Torcida F Actas de las IV Jornadas Internacionales sobre Paleontología de Dinosaurios y su Entorno. Burgos: Salas de los Infantes. pp. 75-90.

[18] Moratalla JJ, Hernán J (2009) Turtle and pterosaur tracks from the Los Cayos dinosaur tracksite, Cameros Basin (Cornago, La Rioja, Spain): tracking the Lower Cretaceous bio-diversity. Revista Española de Paleontología 24 (1): 59-77.

[19] Lockley M, Mickelson DL (1997) Dinosaur and pterosaur tracks in the Summerville and Bluff (Jurassic) beds of eastern Utah and northeastern Arizona. Mesozoic geology and paleontology of the Four Corners region, 48. Guidebook - New Mexico Geological Society pp. 133-138.

[20] Mickelson DL, Lockley MG, Bishop J, Kirkland J (2004) A new pterosaur tracksite from the Jurassic Summerville Formation, near Ferron, Utah. Ichnos 11: 125-142. doi:10.1080/10420940490445437.

[21] Xing L, Harris JD, Gierlinski GD (2011) *Therangospodus* and *Megalosauripus* track assemblage from the Upper Jurassic-Lower Cretaceous Tuchengzi Formation of Chicheng County, Hebei Province, China and their paleoecological implications. Vertebrata PalAsiatica 49(4): 423-434

[22] Fanti F, Contessi M, Nigarov A, Esenov P (2013) New data on two large dinosaur tracksites from the Upper Jurassic of Eastern Turkmenistan (Central Asia). Ichnos 20: 54-71. doi:10.1080/10420940.2013.778845.

[23] Conti MA, Morsilli M, Nicosia U, Sacchi E, Savino V et al. (2005) Jurassic dinosaur footprints from Southern Italy: footprints as indicators of constraints in paleogeographic interpretation. Palaios 20: 534-550. doi:10.2110/palo.2003.p03-99.

[24] Marty D (2008) Sedimentology, taphonomy, and ichnology of Late Jurassic dinosaur tracks from the Jura carbonate platform (Chevenez–Combe Ronde tracksite, NW Switzerland): Insights into the tidal-flat palaeoenvironment and dinosaur diversity, locomotion, and palaeoecology. GeoFocus 21: 1–278.

[25] Pazos PJ, Lazo DG, Tunik MA, Marsicano CA, Fernández DE et al. (2012) Paleoenvironmental framework of dinosaur tracksites and other ichnofossils in Early Cretaceous mixed siliciclastic-carbonate deposits in the Neuquén Basin, northern Patagonia (Argentina). Gondwana Research Available: 10.1016/j.gr.2012.02.003.

[26] Canudo JI, Aurell M, Barco JL, Cuenca-Bescós G, Ruiz-Omeñaca JI (2005) Los dinosaurios de la Formación Villar del Arzobispo (Titónico medio-Berriasiense inferior) en Galve (Teruel). Geogaceta 38: 39-42.

[27] Lockley MG, Wright JL (2001) Trackways of large quadrupedal ornithopods from the Cretaceous: A review. In: Tanke DH Carpenter K Mesozoic vertebrate life. Bloomington and Indianapolis: Indiana University Press pp. 428-442.

[28] Salas R, Guimerà J, Mas R, Martín-Closas C, Meléndez A et al. (2001) Evolution of the Mesozoic central Iberian Rift System and its Cainozoic inversion (Iberian Chain). In: Ziegler PA Cavazza W Robertson AHF Crasquin-Soleau S Peri-Tethys Memoir 6: Peri-Tethyan Rift/Wrench Basins and Passive Margins. Museum National d'Histoire Naturelle, Paris pp. 145–186.

[29] Mas R, García A, Salas R, Meléndez A, Alonso A, et al. (2004) Vera J, editor Geología de España: Sociedad Geológica de España- Instituto Geológico y Minero de España. pp. 503 -509

[30] Gómez Fernández JC, Meléndez N (1994b) Climatic control on Lower Cretaceous sedimentation in a playa-lake system of a tectonically active basin (Huérteles Alloformation, Eastern Cameros Basin, North-Central Spain). Journal of Paleolimnology 11: 91-107. doi:10.1007/BF00683272.

[31] Quijada I, Suárez-González P, Benito MI, Mas JR, Alonso A (2010) Un ejemplo de llanura fluvio-deltaica influenciada por las mareas: el yacimiento de icnitas de Serrantes (Grupo Oncala, Berriasiense, Cuenca de Cameros, N de España). Geogaceta 49: 15-18.

[32] Quijada I, Suárez-González P, Benito MI, Mas JR (2012) Tide-influenced fluvial-deltaic sediments *versus* continental sandy-muddy flat deposits: evidence from the Huerteles Fm (Early Cretaceous, N Spain). In: Tidalites 2012, 8th International Conference on tidal environments, Abstract book: 103-104

[33] Martín-Closas C, Alonso-Millán A (1998) Estratigrafía y bioestratigrafía (Charophyta) del Cretácico Inferior en el sector occidental de la Cuenca de Cameros (Cordillera Ibérica). Revista Sociedad Geológica de España 11 (3–4): 253-269.

[34] Schudack U, Schudack M (2009) Ostracod biostratigraphy in the Lower Cretaceous of the Iberian Chain (eastern Spain). Journal Iberian Geology 35: 141-168.

[35] Aguirrezabala LM, Viera LI (1980) Icnitas de dinosaurios en Bretún (Soria). Munibe 32: 257-279.

[36] Aguirrezabala LM, Viera LI (1983) Icnitas de dinosaurios en Santa Cruz de Yanguas (Soria). Munibe 35: 1-13.

[37] Pascual-Arribas C, Sanz-Pérez E (2000) Icnitas de dinosaurios en Valdelavilla (Soria, España). Estudios Geológicos 56: 41-61.

[38] Fuentes Vidarte C, Meijide Calvo M, Meijide Fuentes F, Meijide Fuentes M (2002) Las huellas de dinosaurios de Castilla y León. In: de Rivero RN Enresa. Patrimonio Geológico de Castilla y León. pp. 360-379.

[39] Fuentes Vidarte C, Meijide Calvo M, Meijide Fuentes F, Meijide Fuentes M (2005) El conjunto faunístico de la base del cretácico inferior de Soria (cuenca de Cameros, grupo Oncala) a través del análisis icnológico. Celtiberia 99: 367-404.

[40] Falkingham PL (2012) Acquisition of high resolution 3D models using free, open-source, photogrammetric software. Palaeontologia Electronica 15: 1–15.

[41] Hammer Ø, Harper DAT, Ryan PD (2001) PAST: Paleontological statistics software package for education and data analysis. Palaeontologia Electronica 4(1): 9.

[42] Manning PL (2004) A new approach to the analysis and interpretation of tracks: examples from the Dinosauria. In: McIlroy D The application of ichnology to palaeoenvironmental and stratigraphic analysis. Geological Society, London, Special Publications 228: 93-123

[43] Milàn J, Bromley RG (2008) The Impact of Sediment Consistency on Track and Undertrack Morphology: Experiments with Emu Tracks in Layered Cement. Ichnos, 15: 18-24.

[44] Jackson SJ, Whyte MA, Romano M (2009) Laboratory-controlled simulations of dinosaur footprints in sand: a key to understanding vertebrate track formation and preservation. Palaios 24: 222-238. doi:10.2110/palo.2007.p07-070r.

[45] Falkingham PL, Bates KT, Margetts L, Manning PL (2011) The 'Goldilocks' effect: preservation bias in vertebrate track assemblages. J R Soc Interface 8: 1142-1154. doi:10.1098/rsif.2010.0634. PubMed: 21233145. 21233145 PMC3119880

[46] Díaz-Martínez I, Pérez-Lorente F, Canudo JI, Pereda-Suberbiola X (2012) An ichnotaxonomical view of the large ornithopod footprints. Fundamental 20: 63-64.

[47] Lockley MG, Santos VF, Meyer C, Hunt A (1998) A new dinosaur tracksite in the Morrison Formation, Boundary Butte, Southeastern Utah. In: Carpenter K Chure D Kirkland K The Upper Jurassic Morrison Formation: An interdisciplinary study. Modern. Geology 23 (2): 317-330

[48] Sarjeant WA, Delair JB, Lockley MG (1998) The footprints of *Iguanodon*: a history and taxonomic study. Ichnos 6 (3): 183-202. doi:10.1080/10420949809386448.

[49] Meyer CA, Thüring B (2003) The first iguanodontid dinosaur tracks from the Swiss Alps (Schrattenkalk Formation, Aptian). Ichnos 10: 221-228. doi:10.1080/10420940390256186.

[50] Kim JY, Lockley MG, Kim HM, Lim JD, Kim KS (2009) New dinosaur tracks from Korea, *Ornithopodichnus* *masanensis* ichnogen. et ichnosp. nov (Jindong Formation, Lower Cretaceous): implications for polarities in ornithopod foot morphology. Cretaceous Research 30: 1387-1397. doi:10.1016/j.cretres.2009.08.003.

[51] Lockley MG, Huh M, Kim BS (2012) *Ornithopodichnus* and Pes-Only Sauropod Trackways from the Hwasun Tracksite, Cretaceous of Korea. Ichnos 19 (1-2): 93-100. doi:10.1080/10420940.2011.625726.

[52] Lockley MG, Meyer CA, dos Santos VF (1998) *Megalosauripus* and the problematic concept of Megalosaur footprints. GAIA 15: 312-337.

[53] Pascual-Arribas C, Hernández-Medrano N, Latorre-Macarrón P, Sanz-Pérez E (2009) El icnogénero *Iguanodontipus* en el yacimiento de "Las Cuestas I" (Santa Cruz de Yanguas, Soria, España). Studia Geologica Salmanticensia 45 (2): 105-128.

[54] Lockley MG, Wright JL, Thies D (2004) Some observations on the dinosaur tracks at Münchenhagen (Lower Cretaceous), Germany. Ichnos 11: 262-274.

[55] Diedrich C (2004) New important iguanodontid and theropod trackways of the tracksite Obernkirchen in the Berriasian of NW Germany and megatracksite concept of Central Europe. Ichnos 11: 215-228. doi:10.1080/10420940490444924.

[56] Wings O, Broschinski A, Knötschke N (2005) New theropod and ornithopod dinosaur trackways from the Berriasian of Münchehagen (Lower Saxony, Germany). Kaupia, Darmstädter Beiträge zur Naturgeschichte 14: 105.

[57] Wright JL, Barrett PM, Lockley MG, Cook E (1998) A review of the Early Cretaceous Terrestrial Vertebrate Track-Bearing Strata of England and Spain. Lower and Middle Cretaceous Terrestrial Ecosystems. In: Lucas SG Kirkland JI Estep JW Bulletin of the; New Mexico Museum of Natural History and Science Lower and Middle Cretaceous Terrestrial. Ecosystems 14: 143-153.

[58] Wright JL (1999) Ichnological evidence for the use of the forelimb in iguanodontid locomotion. In: Unwin D Cretaceous fossil vertebrates. Special Papers in Palaeontology 60: 209-212.

[59] Woodhams KE, Hines JS (1989) Dinosaur footprints from the Lower Cretaceous of East Sussex, England. In: Gillette DD Lockley MG Dinosaur Tracks and Traces. Cambridge University Press pp. 299-307.

[60] Goldring R, Pollard JE, Radley JD (2005) Trace fossils and pseudofossils from the Wealden strata (non-marine Lower Cretaceous) of southern England. Cretaceous Research 26: 665-685. doi:10.1016/j.cretres.2005.03.001.

[61] Moratalla JJ, Sanz JL, Jiménez S, Lockley MG (1992) A quadrupedal ornithopod trackway from the Lower Cretaceous of La Rioja (Spain): inferences on gait and hand structure. Journal of Vertebrate Paleontology 12: 150-157. doi:10.1080/02724634.1992.10011445.

[62] Moratalla JJ, Sanz JL, Jiménez S (1994) Dinosaur tracks from the Lower Cretaceous of Regumiel de la Sierra (province of Burgos, Spain): inferences on a new quadrupedal ornithopod trackway. Ichnos 3: 89-97. doi:10.1080/10420949409386376.

[63] Santos VF, Callapez PM, Rodrigues NPC (2012) Dinosaur footprints from the Lower Cretaceous of the Algarve Basin (Portugal): New data on the ornithopod palaeoecology and palaeobiogeography of the Iberian Peninsula. Cretaceous Research: 1-12.27087716

[64] Cobos A, Gascó F (2012) Presencia del icnogénero *Iguanodontipus* en el Cretácico Inferior de la provincia de Teruel (España). Geogaceta 52: 185-188.

[65] Herrero-Gascón J, Pérez-Lorente F (2013) Nuevas aportaciones icnológicas de Galve (Teruel, España). Grandes huellas ornitópodas En El yacimiento de Santa Bárbara Geogaceta 53: 21-24.

[66] Lockley MG (1987) Dinosaur Footprints from the Dakota Group of Eastern Colorado. Mountain Geologist 24: 107-122.

[67] Paul G. 1991 The many myths, some old, some new of dinosaurology. Modern Geology, 16- 69 -99

[68] McDonald AT (2012) Phylogeny of Basal Iguanodonts (Dinosauria: Ornithischia): An. Update - PLOS ONE 7(5): e36745. doi:10.1371/journal.pone.0036745. 22629328 PMC3358318

[69] Moreno K, Carrano MT, Snyder R (2007) Morphological changes in pedal phalanges through ornithopod dinosaur evolution: A biomechanical approach. J Morphol 268 (1): 50-63. doi:10.1002/jmor.10498. PubMed: 17146773. 17146773

[70] Maidment SCR, Barrett PM (In press) Osteological correlates for quadrupedality in ornithischian dinosaurs. Acta Paleontological Polonica.

